# *RAC1* Alterations Induce Acquired Dabrafenib Resistance in Association with Anaplastic Transformation in a Papillary Thyroid Cancer Patient

**DOI:** 10.3390/cancers13194950

**Published:** 2021-09-30

**Authors:** Rozita Bagheri-Yarmand, Naifa L. Busaidy, Elena McBeath, Brian P. Danysh, Kurt W. Evans, Tyler J. Moss, Argun Akcakanat, Patrick K. S. Ng, Christina M. Knippler, Jalyn A. Golden, Michelle D. Williams, Asha S. Multani, Maria E. Cabanillas, Kenna R. Shaw, Funda Meric-Bernstam, Manisha H. Shah, Matthew D. Ringel, Marie Claude Hofmann

**Affiliations:** 1 Department of Endocrine Neoplasia and Hormonal Disorders, University of Texas MD Anderson Cancer Center, Houston, TX 77030, USA; ryarmand@mdanderson.org (R.B.-Y.); nbusaidy@mdanderson.org (N.L.B.); emfujiwara@mdanderson.org (E.M.); bdanysh@broadinstitute.org (B.P.D.); jalyn.golden@aggienetwork.com (J.A.G.); mcabani@mdanderson.org (M.E.C.); 2 Department of Investigative Cancer Therapeutics, University of Texas MD Anderson Cancer Center, Houston, TX 77030, USA; kwevans@mdanderson.org (K.W.E.); aakcakanat@mdanderson.org (A.A.); patrick.ng@jax.org (P.K.S.N.); krshaw@mdanderson.org (K.R.S.); fmeric@mdanderson.org (F.M.-B.); 3 Bioinformatics & Computational Biology, University of Texas MD Anderson Cancer Center, Houston, TX 77030, USA; TylerMoss@viracor-eurofins.com; 4 Division of Endocrinology, Diabetes, and Metabolism, Department of Internal Medicine, The Ohio State University, Columbus, OH 43210, USA; christina.michelle.knippler@emory.edu (C.M.K.); matthew.ringel@osumc.edu (M.D.R.); 5 Department of Hematology and Medical Oncology, Emory University Winship Cancer Institute, Atlanta, GA 30322, USA; 6 Department of Pathology, University of Texas MD Anderson Cancer Center, Houston, TX 77030, USA; mdwillia@mdanderson.org; 7 Department of Genetics, University of Texas MD Anderson Cancer Center, Houston, TX 77030, USA; amultani@mdanderson.org; 8 Division of Medical Oncology, Department of Internal Medicine, The Ohio State University, Columbus, OH 43210, USA; manisha.shah@osumc.edu

**Keywords:** papillary thyroid carcinoma, anaplastic thyroid carcinoma, BRAF, RAC1, PAK1, aneuploidy, kinase inhibitors, drug resistance

## Abstract

**Simple Summary:**

We identified an acquired *RAC1* (P34R) mutation in the metastatic tumor of a BRAF-mutated papillary thyroid cancer patient treated with the BRAF inhibitor dabrafenib. Further investigations uncovered a high *RAC1* copy number in the metastatic sample and its derived cell line. We demonstrated that an increase in *RAC1* copy numbers could lead to increased tumor cell growth independently of the *RAC1* (P34R) mutation. Further, we identified polyploidy of chromosome 7 in the metastatic sample and derived cell line, which accounted for amplification of other genes, their increased expression and therefore their ability to also induce resistance to therapy.

**Abstract:**

*BRAF*-activating mutations are the most frequent driver mutations in papillary thyroid cancer (PTC). Targeted inhibitors such as dabrafenib have been used in advanced *BRAF*-mutated PTC; however, acquired resistance to the drug is common and little is known about other effectors that may play integral roles in this resistance. In addition, the induction of PTC dedifferentiation into highly aggressive *KRAS*-driven anaplastic thyroid cancer (ATC) has been reported. We detected a novel *RAC1* (P34R) mutation acquired during dabrafenib treatment in a progressive metastatic lesion with ATC phenotype. To identify a potential functional link between this novel mutation and tumor dedifferentiation, we developed a cell line derived from the metastatic lesion and compared its behavior to isogenic cell lines and primary tumor samples. Our data demonstrated that *RAC1* mutations induce changes in cell morphology, reorganization of F-actin almost exclusively at the cell cortex, and changes in cell adhesion properties. We also established that *RAC1* amplification, with or without mutation, is sufficient to drive cell proliferation and resistance to *BRAF* inhibition. Further, we identified polyploidy of chromosome 7, which harbors *RAC1*, in both the metastatic lesion and its derived cell line. Copy number amplification and overexpression of other genes located on this chromosome, such as *TWIST1*, *EGFR*, and *MET* were also detected, which might also lead to dabrafenib resistance. Our study suggests that polyploidy leading to increased expression of specific genes, particularly those located on chromosome 7, should be considered when analyzing aggressive thyroid tumor samples and in further treatments.

## 1. Introduction

Thyroid carcinoma is the most common endocrine malignancy. In the United States, it accounts for 4% of all cancers in women [1]. The majority of thyroid malignancies are derived from the follicular cells of the thyroid gland and include papillary (PTC) and follicular carcinoma (FTC) [2]. Approximately 85% of these tumors are successfully treated with thyroidectomy followed by radioactive iodine or thyroid-stimulating hormone suppression therapy in selected cases [3]. However, about 10–20% of patients will develop advanced metastatic disease, and many will not respond to conventional therapy [4]. In PTC the most frequent driver mutations (50–60%) occur in the *B-RAF* (*BRAF*) gene, which encodes a member of the RAF family of serine/threonine kinases [5]. Mutations in the kinase domain of this molecule, such as *BRAF* (V600E), *BRAF* (K601N), or *BRAF* (T599del) result in constitutively activated catalytic activity and subsequent activation of downstream kinases along the MAPK pathway [6,7], causing unrestricted cell proliferation. *BRAF*-mutated tumor cells are sensitive to V600E-specific inhibitors (vemurafenib and dabrafenib) [8,9,10,11], with dabrafenib currently being used in clinical trials for advanced metastatic disease in PTCs [12]. However, even in combination with inhibitors targeting downstream effectors (trametinib, a MEK inhibitor), acquired resistance to the drug is nearly universal and the disease progresses [13].

Acquired resistance to BRAF inhibitors presents a significant therapeutic challenge to the long-term survival of affected patients, and our modest understanding of how PTC tumors escape inhibitory controls limits the selection of optimal second-line treatments. We previously described acquired resistance to the BRAF inhibitor vemurafenib in a *BRAF* (V600E)-mutated PTC cell line through de novo acquisition of a *KRAS* (G12D) activating mutation following five months of in vitro treatment with the drug [14]. *BRAF* and *KRAS* mutations are mutually exclusive in untreated primary thyroid cancers [5]. However, we postulated that exposure to the BRAF inhibitor provides evolutionary pressure that permits the emergence of the concomitant *KRAS* mutant clone and co-expression of the molecules. Supporting this view, we and others recently identified secondary *KRAS* and *NRAS* mutations in several patients with *BRAF* (V600E)-mutated PTCs that progressed during treatment with BRAF inhibitors [15,16]. In the present study, through whole-exome sequencing (WES), we identified the clonal acquisition of a novel *RAC1* (P34R) mutation in the recurrent tumor of a PTC patient (BRAF^T599del;K601N^) that progressed under dabrafenib treatment. RAC1 is a member of the RAS-related C3 botulinum toxin substrate (RAC) subfamily of RHO family small guanosine triphosphatases (GTPases) within the rat sarcoma (RAS) superfamily of GTPases. Small GTPases are activated by transitioning between GDP-bound (inactive) and GTP-bound (active) states, are pleiotropic regulators of the cell cycle, cell adhesion and motility, and have been implicated in tumorigenesis. Specifically, RAC1 is involved in the regulation of cytoskeleton reorganization, cell survival, cell–cell adhesion, and motility through the ACTB network [17]. This emergent mutation (P34R) differs from other *RAC1* resistance mutations so far found in melanoma or other tumor types, e.g., *RAC1* (P29S) [18,19,20,21].

In the current study, we used a PDX-derived cell line and other PTC cell lines to determine whether *RAC1* alterations were capable of driving growth of PTC cells and enabling their resistance to BRAF (V600E) specific inhibitors. The patient sample at progression and the PDX-derived cell line exhibited a clear dedifferentiated phenotype with anaplastic thyroid cancer (ATC) features. Inhibition of RAC1 activity led to the re-acquisition of dabrafenib sensitivity. Further studies demonstrated polyploidy of chromosome 7 in the patient sample at progression and the derived cell line, as well as additional gene amplification, which accounted for an increase in mutated *RAC1* copy number and gene expression. Thus, RAC1 overexpression, perhaps influenced by the mutation, might explain resistance to dabrafenib as well as cell growth and invasion in three-dimensional spheroid cultures. These findings provide additional critical insights, and a role for *RAC1* copy number variation, in acquired resistance to BRAF inhibitors in *BRAF*-mutated cancers.

## 2. Results

### 2.1. A Secondary RAC1(P34R) Mutation Is Acquired in PTC following Dabrafenib Treatment

Our index patient is a 53-year-old male with PTC who was incidentally found to have lung nodules on chest X-ray performed for other clinical reasons. Biopsy of the most accessible lung lesion revealed PTC. He then underwent a total thyroidectomy and neck dissection, and was found to have a 6.5 cm PTC with extrathyroidal extension and metastases to multiple lateral neck lymph nodes. He then underwent radioactive iodine treatment and the post-treatment scan showed thyroid bed uptake and no iodine avid lung metastases. He was subsequently treated with thyroid-stimulating hormone suppression therapy and received three more treatments of radioactive iodine over time with no anatomic response, but there was a modest reduction in serum thyroglobulin. He underwent external beam radiotherapy of part of each of the bilateral upper lobes of his lungs for progressive disease in two separate settings. He was referred to The University of Texas MD Anderson Cancer Center at which time he was noted to have respiratory symptoms from previous therapies and disease progression in his lungs over the previous year. Due to disease progression and lack of response to radioactive iodine therapy, systemic therapy was proposed. To best determine treatment options, DNA was isolated from a FFPE sample (neck dissection) and sent for mutational testing using an internal CLIA-approved 50-gene next generation sequencing (NGS) panel (Table S1). A *BRAF* (A598_T599delinsA) and a *BRAF* (K601N) mutation were identified, as well as an ataxia-telangiectasia-mutated (*ATM*) gene mutation (*ATM* (P604S)). Given the presence of the BRAF mutation, the patient was enrolled in a NCCN sponsored randomized controlled trial investigating dabrafenib versus dabrafenib in combination with trametinib in *BRAF* mutated thyroid cancer (NCT01723202). The patient was randomized into the dabrafenib single agent cohort. After two months of treatment, he was restaged and noted to have a decrease in the size of his multiple neck and upper mediastinal nodes. However, large progressive disease in a prevascular mediastinal node was also observed. Additional biopsies of the site of progressive disease were taken under the MD Anderson Unusual Responder clinical trial (NCT01772771), where tumors are assessed using whole exome sequencing (WES) to provide options for personalized cancer therapy.

A fine needle aspirate (FNA) of the large mediastinal lymph node at the time of progression was analyzed by WES for more comprehensive testing (Table 1). In addition, two pre-treatment biopsies (from a soft tissue lesion and a cervical lymph node) were characterized using the MD Anderson targeted T200.1 panel (Table S2). All samples harbored the BRAF (A598_T599delinsA) mutation resulting in an in-frame deletion of Thr at position 599. The pre-treatment cervical lymph node and the metastatic mediastinal lymph node harbored an additional A > T substitution causing a *BRAF* (K601N) missense mutation. A *RAC1* (P34R) mutation, predicted to be activating, was identified only in the metastatic mediastinal lymph node with an allele frequency of 25.6% (Table 1). While both platforms use NGS and amplified RAC1 for analysis, T200.1 and WES assays are not identical. However, because the allelic frequency of the mutant was internally controlled versus wild-type, and the difference in allele frequency (0% vs. 25%) was very large, we are confident that the *RAC1* mutation appeared during dabrafenib treatment. *RAC1* mutations such as P29S have been shown to cause resistance to BRAF inhibitors in melanoma [22,23], but the consequences of a P34R mutation are unknown. Contrary to some primary melanoma, that can harbor both *BRAF* and *RAC1* mutations simultaneously, these mutations are not concomitantly found in papillary thyroid cancer according to TCGA (analyzed using cBioPortal). Other mutations found in the pre- and post-treatment tumors appear to be subclonal and are shown in Table 1. We established a cell line, PDX.008.CL, from a mouse xenograft derived from the mediastinal nodal sample at progression. The *RAC1* (P34R) mutation was confirmed in the PDX.008.CL line following 20 passages (Sanger sequencing, Figure S1a). The BRAF mutations were confirmed using NGS (Figure S1b).

### 2.2. Tumor Histopathology Indicated an Anaplastic Phenotype at Progression

**Figure 1:**
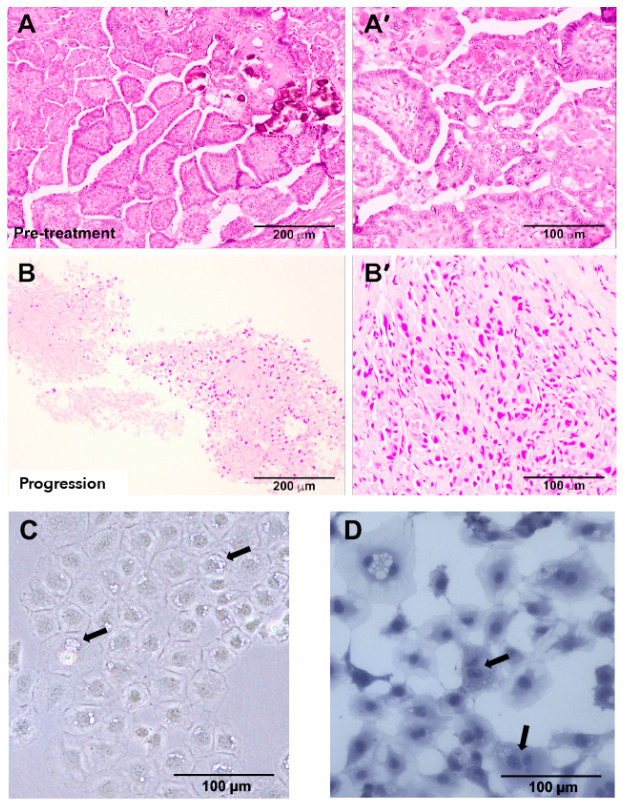
Samples histology of a PTC patient before and after dabrafenib treatment, and morphology of a derived cell line. (**A**) H&E preparation of a pretreatment sample showing papillary thyroid cancer histology. (**A’**) Enlargement of A showing cells organized in papillae. Cells present with enlarged nuclei with irregular contours, intranuclear inclusions, and pale chromatin. (**B**) Overall histology of a mediastinal metastasis sample at progression (FNA, H&E preparation). (**B’**) Enlargement of B showing cells distributed without an organized pattern. Cells have a spindle or round cell morphology resembling poorly differentiated (PDTC) or anaplastic (ATC) thyroid cancer. (**C**) Phase contrast micrograph showing the cobblestone arrangement of PDX.008.CL cells in culture. Cells are mostly round or cuboidal with round nuclei containing prominent nucleoli. The cytoplasm also contains prominent vacuoles (arrows). (**D**) Hematoxylin staining of PDX.008.CL cells in culture showing multinucleated cells (arrows).

The patient’s original tumor exhibited a typical differentiated PTC morphology characterized by the presence of neoplastic papillae frequently branched and coated by one layer of cubic epithelial cells (Figure 1A,A’). Tumor samples at progression exhibited a high-grade PTC or anaplastic thyroid cancer (ATC) morphology, with cellular discohesion and hyperchromatic or polymorphic nuclei, and areas of necrosis (Figure 1B,B’). Phase-contrast microscopy of cultured PDX.008.CL cells indicated the presence of round to cuboidal cells with cobblestone morphology (Figure 1C) and accumulation of vacuoles (arrows). Multinucleated cells were also apparent by hematoxylin staining (Figure 1D, arrows).

### 2.3. Expression of Mutated BRAF and RAC1 Proteins Increases Cell Growth

**Figure 2:**
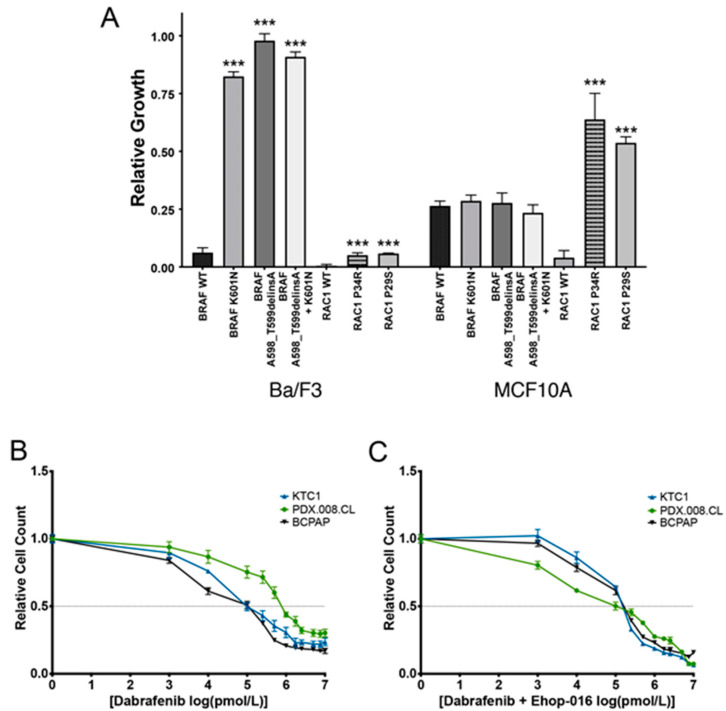
Effects of BRAF and RAC1 mutations on the proliferation of different cell types. (**A**) The effects of the BRAF (A598_T599delinsA) and (K601N) mutations separately or combined, as well as the effects of the RAC1(P34R) and RAC1(P29S) mutations on the growth of murine Ba/F3 pro-B cells and human MCF10A normal breast epithelial cells were tested in culture. Cells were transiently transfected with lentiviral expression vectors and cultured without the addition of growth factors (interleukin 3 for Ba/F3 and EGF for MCF10A cells). Data indicated that the BRAF mutations significantly affected the growth of lymphoid cells while the RAC1 mutations significantly affected the proliferation of the breast epithelial cells. *n* = 3, *** = *p* < 0.005. (**B**) The effect of dabrafenib on the proliferation of KTC1, BCPAP, and PDX.008.CL cells was evaluated in a dose–response assay. The half maximal inhibitory concentration (IC50) value for dabrafenib was 0.1 µM for both KTC1 and BCPAP cells, while the IC50 value was 1 µM for the PDX.008.CL cell line, indicating a 10-fold resistance to dabrafenib of this cell line in comparison to the other lines. (**C**) The effect of the RAC1 activity inhibitor EHop-016 in combination with dabrafenib on KTC1, BCPAP and PDX.008.CL cells was evaluated. EHop-016 restored the dabrafenib sensitivity of PDX.008.CL cells back to the levels of KTC1 and BCPAP cells (IC50 = 0.1 µM), indicating that the RAC1 mutation had a major effect on dabrafenib resistance.

To investigate the ability of BRAF (A598_T599delinsA), BRAF (K601N), and RAC1(P34R) proteins to increase cell proliferation, we performed cell viability assays using transient transductions of the mutated *BRAF* and *RAC1* genes into cell lines often used to screen oncogenic drivers [24]. As a positive control, we transduced the gene coding for the known *RAC1* (P29S) mutation [18]. Ba/F3 cells (murine, IL-3 dependent, hematopoietic cells) and MCF10A cells (human, EGF-dependent, mammary epithelial cells) expressing mutant BRAF and RAC1 proteins were grown without essential growth factors. BRAF K601N and T599del proteins, alone and in combination induced strong cell growth in Ba/F3 cells but had no functional effect in MCF10A cells, compared to BRAF WT (Figure 2A). The RAC1 P34R and P29S proteins displayed a small but significant functional effect in Ba/F3 cells compared to wild-type RAC1. In comparison, both RAC1-mutated proteins strongly and significantly increased cell proliferation in MCF10A cells. These results demonstrate that RAC1 (P34R) can drive cell growth in vitro to the same extent as RAC1 (P29S) in mammary epithelial cells.

### 2.4. Concurrent BRAF and RAC1 Alterations Drive BRAF Inhibitor Resistance

We next sought to determine if acquisition of a *RAC1* mutation resulted in dabrafenib resistance in the PDX.008.CL cells, similar to the patient’s metastatic mediastinal tumor at progression. We therefore treated BRAF (V600E)-mutated PTC cells (KTC1, BCPAP) and PDX.008.CL cells with different concentrations of inhibitors targeting RAC1 (EHop-016) and BRAF (V600E) (dabrafenib). As shown in Figure 2B, PDX.008.CL cell proliferation was affected by dabrafenib, but the cells were significantly more resistant to this inhibitor (IC_50_ value of 1.0 μM) compared to KTC1 and BCPAP cells (IC_50_ values of 0.1 µM) (*p* < 0.0001). In contrast, the PDX.008.CL cells displayed much greater sensitivity to the combination of dabrafenib with a RAC1 activity inhibitor (EHop-016) with a ten-fold decrease in IC_50_ value from 1 µM to 0.1 µM, while the sensitivity of both KTC1 and BCPAP cells did not change (Figure 2C). These results indicated that increased RAC1 activity might be responsible for the resistance of the PDX.008.CL cells to dabrafenib.

### 2.5. RAC1 mRNA and RAC1(P34R) Protein Are Over-Expressed in PDX.008.CL Cells in Comparison to Other BRAF-Mutated PTC Cells

**Figure 3:**
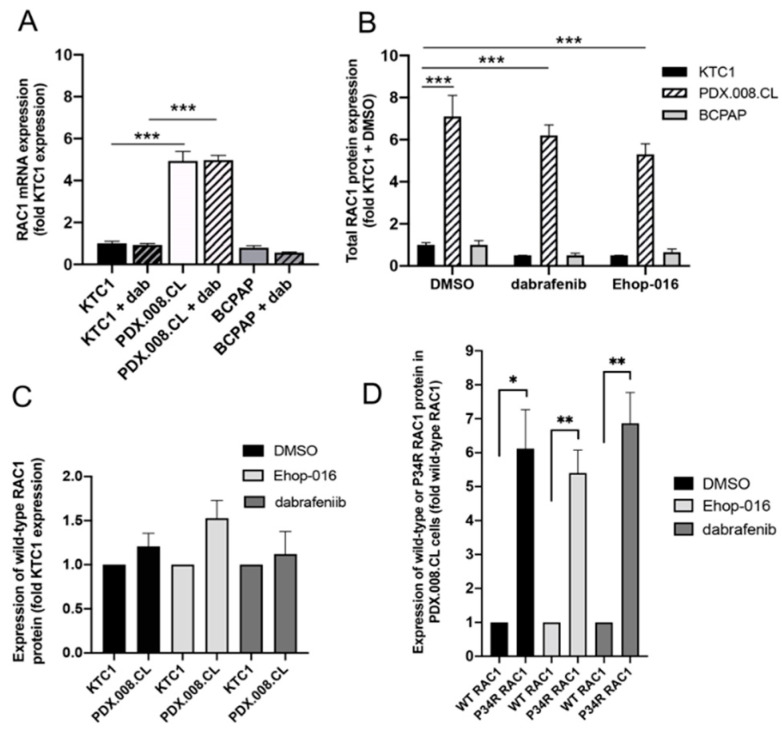
RAC1 mRNA and protein expression/activity in PDX.008.CL cells in comparison to other thyroid cancer cell lines. (**A**) RAC1 total mRNA expression in the PDX.008.CL cell population was 5-fold that of KTC1 and BCPAP cells (*n* = 3, *** = *p*< 0.005). (**B**) RAC1 total protein expression in the PDX.008.CL cells was about 5–7-fold that of KTC1 and BCPAP cells. RAC1 protein expression was not altered by dabrafenib nor EHop-016, an inhibitor of RAC1 activity (*n* = 3, *** = *p* < 0.005). (**C**) The level of expression of wild-type RAC1 protein in PDX.008.CL cells was not significantly different from that in KTC1 cells (*n* = 4, *p* = 0.19). (**D**) In PDX.008.CL cells the level of expression of RAC1 (P34R) protein was 6-fold that of wild-type RAC1, and was not altered by dabrafenib nor EHop-016, a specific inhibitor of RAC1 activity (*n* = 5, * = *p* < 0.05, **= *p* < 0.005).

In order to understand more in depth the effects of the RAC1 mutation, we assessed the expression levels of *RAC1* wild-type and *RAC1* (P34R) mRNA and proteins in these cells and compared them with other PTC cell lines such as KTC1 and BCPAP, which are both *BRAF*-mutated. As seen in Figure 3A,B and Figure S3, expression of total *RAC1* mRNA and RAC1 protein are significantly increased in PDX.008.CL cells in comparison to other PTC cell lines. By using a combination of antibodies recognizing different epitopes (Table S3), we revealed that the amount of wild type RAC1 protein expressed in PDX.008.CL is not significantly different from that in KTC1 cells (Figure 3C), while the amount of expressed RAC1(P34R) protein is significantly higher (~6 times) in the PDX.008.CL cells (Figure 3D and Figure S4). As expected, neither dabrafenib nor EHop-016 significantly influenced the amount of RAC1 expressed in these cells (Figure 3B,D).

### 2.6. The PDX.008.CL Cells Are Aneuploid

**Figure 4:**
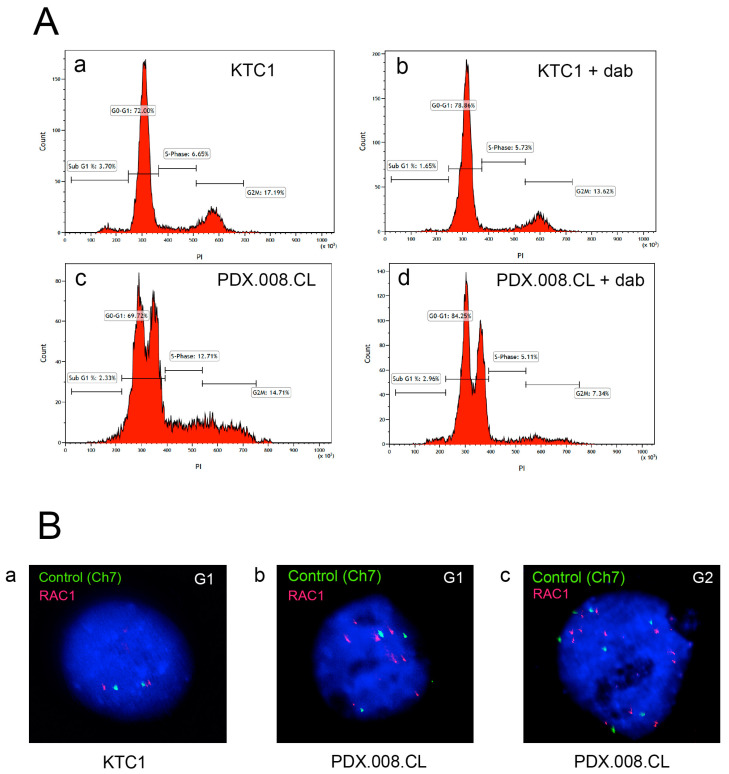
PDX.008.CL cells are aneuploid. (**A**) KTC1 (**a**,**b**) and PDX.008.CL cells (**c**,**d**) were analyzed by flow cytometry. KTC1 cells were diploid, showing 2N DNA at G0/G1 and 4N DNA at G2 (**a**) while PDX.008.CL cells were aneuploid, showing 2N DNA and an additional peak at G0/G1 (**c**). Dabrafenib treatment (0.1 µM dabrafenib for 2 days) induced cell cycle arrest in both cell lines (**b**,**d**) as seen by an increase in the percentage of diploid cells in G0/G1. Dabrafenib reduced the percentage of aneuploid cells by ~20% (**d**). Count = cell number; PI = propidium iodide (DNA amount). (**B**) Fluorescence in situ hybridization (FISH) of KTC1 and PDX.008.CL cells with a *RAC1* probe (red) and a chromosome 7 centromeric probe (green) as control. The KTC1 cell line is diploid for chromosome 7 (**a**), while the PDX.008.CL line shows chromosome 7 trisomy and additional *RAC1* duplications (**b**,**c**).

To understand the cause of the high RNA and protein expression of RAC1, and because the PDX.008.CL cells exhibited a multinucleated morphology (Figure 1D), we analyzed KTC1 and PDX.008.CL cells by flow cytometry, and examined the effects of dabrafenib treatment on cell cycle progression. Figure 4A demonstrates that the KTC1 cells were diploid and that dabrafenib at a concentration of 0.1 µM induced G0/G1 arrest after 48 h exposure, with an increase in the number of cells in G0/G1 from 72% to 79%, and a decrease in the number of cells in G2 from 17% to 13.6% (Figure 4A(a,b). The PDX.008.CL cell line exhibited two distinct G0/G1 peaks consistent with polyploidy (Figure 4A(c,d)). In this cell line, the dabrafenib treatment increased the percentage of cells in G0/G1 (Figure 4A(d)) from 70% to 84%. Cell cycle arrest mainly affected the diploid cells subpopulation. Dabrafenib markedly decreased the percentage of cells in S and G2 phase. These results confirm the cytostatic, but not cytotoxic, effects of kinase inhibitors. We then used interphase FISH to evaluate *RAC1* copy numbers at G0/G1 and G2 in KTC1 and PDX.008.CL cells (Figure 4B). A chromosome 7 centromeric probe (RAC1 is located on chromosome 7) was used as control. Figure 4B(a) shows that the KTC1 cell line is diploid for chromosome 7 and *RAC1* (2 centromeric alleles and 2 RAC1 alleles at G0/G1). In comparison, the PDX.008.CL cells show trisomy of chromosome 7 (3 centromeric alleles at G0/G1 and 6 centromeric alleles at G2) (Figure 4B(b,c)). Interestingly 6 copies (G0/G1) and 12 copies (G2) of RAC1 could be detected in the PDX.008.CL cells that could be explained by trisomy 7 and additional distal duplication of 7p since RAC1 is located at 7p22.1.

### 2.7. RAC1 Gene Copy Number Is Increased in the Patient’s Resistant Tumor Tissue and PDX.008.CL Cells

**Figure 5:**
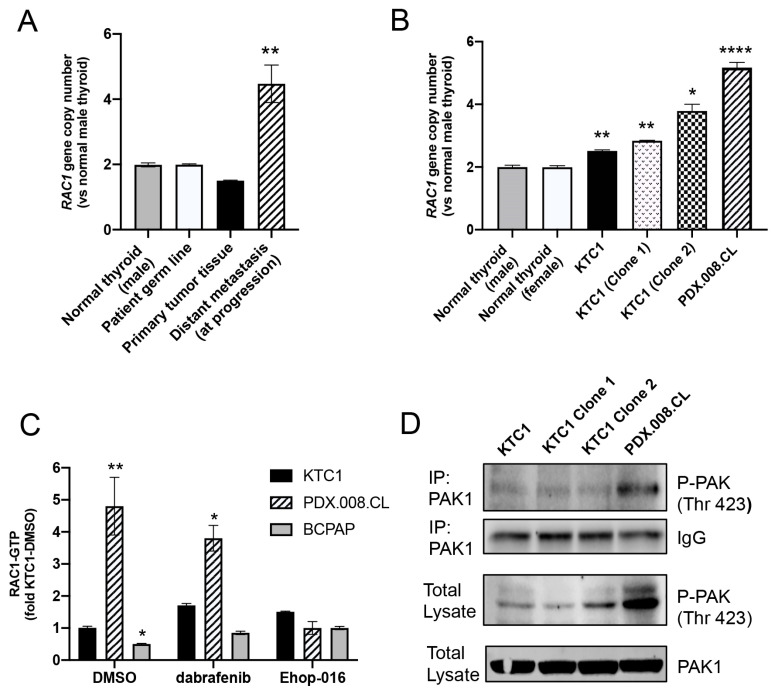
RAC1 copy numbers in patient samples and cell lines and RAC1/PAK1 activity. (**A**) RAC1 gene copy number in the PTC patient tumor samples (primary tumor and metastatic lesion) indicating polyploidy of RAC1 in the metastatic sample at progression in comparison with normal male thyroid, patient germline control and primary tumor tissue. An average of 5 copies of RAC1 was found in the metastatic sample at progression. (*n* = 3, ** = *p* <0.005). (**B**) RAC1 gene copy number in unsynchronized KTC1 and PDX.008.CL cells in comparison to male and female normal thyroid controls. An average of five copies were detected in the PDX.008.CL cells, and 2–4 copies in the KTC1 clones. (*n* = 3, * = *p* <0.05, ** = *p* < 0.005, **** = *p* < 0.0001). (**C**) Total activity of RAC1 protein in different cell types was measured by quantifying its binding to GTP. The activity of RAC1 was significantly elevated in PDX.008.CL cells in comparison to the other thyroid cancer cell types KTC1 and BCPAP. Further, RAC1 activity in PDX.008.CL cells was reduced down to levels comparable to the other cell types by using EHop-016. (*n* = 3, * = *p* < 0.05, ** = *p* < 0.01). (**D**) Increased activity of PAK1 in PDX.008.CL cells was detected after immunoprecipitation using a PAK1 antibody followed by Western blot detection of phospho-PAK1 at Thr423. PAK1 activity was also higher in KTC1 clone 2 cells than in KTC1 clone 1 and KTC1 control cells (total lysates).

We next quantified the average copy number of the *RAC1* gene in the patient tumor samples before treatment and at progression. Normal male and female blood samples were also analyzed as references. Results showed that the primary cancer tissue sample of the patient harbored an average of less than two copies of the *RAC1* gene, and the resected mediastinal metastasis with resistance and growth on dabrafenib had four copies on average (Figure 5A). This indicated that genomic amplification, alone or in addition to the P34R mutation, might have caused progression under dabrafenib treatment. Figure 5B shows that in the unsynchronized PDX.008.CL cell population, the average *RAC1* gene copy number per cell was 5.0 ± 0.16 and was significantly higher than KTC1 cells (2.5 ± 0.03) or the normal human samples (2.0 ± 0.05). In order to understand the functional consequences of *RAC1* amplification alone, we generated KTC1 PTC clones harboring additional *RAC1* wild-type copies. The KTC1 clone 1 harbored about 3 copies of *RAC1* (2.8 ± 0.015) while the KTC1 clone 2 harbored about 4 copies (3.8 ± 0.22) of *RAC1* (Figure 5B).

### 2.8. PDX.008.CL Cells Show High RAC1 and PAK1 Activation

We next sought to determine whether the PDX.008.CL cells contained higher levels of GTP-bound (active) RAC1 using a PAK1-binding domain (PBD) pull down assay. Active RAC1 was immunoprecipitated from KTC1, PDX.008.CL, and BCPAP cells using a GST-PAK1-PBD fusion protein that strongly binds GTP-bound RAC1, then probed for total RAC1. Endogenous levels of GTP-bound RAC1 were significantly higher (4.9-fold) in the PDX.008.CL cells but significantly lower in the BCPAP cells compared to KTC1 cells (Figure 5C and Figure S5). As expected, dabrafenib did not significantly change the levels of RAC1 protein activity, while activity was inhibited by the RAC1 inhibitor EHop-016.

Because RAC1 mediates its effects mainly through activation of the p21-activated kinase (PAK1-3) [25], we also examined the activity of PAK1 in PDX.008.CL cells and KTC1 clones. It has been shown also that PAK1 hyperactivity leads to centrosome amplification and promotes an abnormal organization of mitotic spindles [26]. Further, PAK1 is important for *BRAF*-mutated thyroid cancer cell growth and invasion [27,28,29]. Figure 5D and Figure S6 show phosphorylation of PAK1 at Thr423, which is required for its activation after interaction with activated RAC1 [30,31]. This experiment demonstrated that PAK1 activation is higher in PDX.008.CL cells than in KTC1 control cells, and also higher in KTC1 clone 2 cells than in KTC1 clone 1 and KTC1 control cells (total lysates). Increased PAK1 activation might therefore be due to both an increase in RAC1 expression and activation.

### 2.9. Increased RAC1 Copy Numbers Induce Morphological and Growth Changes

**Figure 6:**
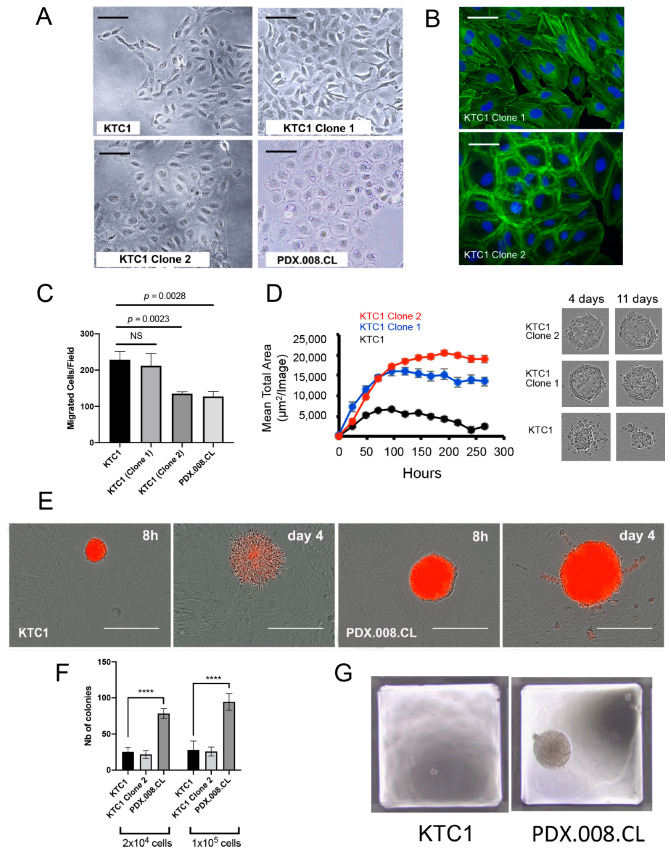
Effects of RAC1 gene amplification in KTC1 subclones. (**A**) There were noticeable shape differences between the original KTC1 cells and KTC1 cells Clone 2 harboring 4 RAC1 copies. In particular, similar to the PDX.008.CL cells, the KTC1 Clone 2 cells were round and flat and showed an increase in perinuclear vacuoles (bars = 50 µm). (**B**) KTC1 cells Clone 1 showed the presence of actin stress fibers (green fluorescence) and a limited amount of cortical actin, while KTC1 Clone 2 showed striking actin reorganization into thick cortical actin bundles (bars = 20 µm). (**C**) Migration assay data for KTC1 clones and PDX.008.CL cells demonstrated that RAC1 amplifications significantly impaired cell motility in 2D cultures (*n* = 6). (**D**) KTC1 spheroid sizes significantly increased with RAC1 gene copy numbers as quantified with the IncuCyte platform (3D cultures, media without Matrigel). Phase contrast micrographs showed that control KTC1 spheroids broke apart after 4 days in culture while spheroids with excess RAC1 grew and remained stable for another 7 days. (**E**) KTC1 and PDX.008.CL cells were grown as spheroids in 3D Matrigel cultures. After 4 days, KTC1 cells distinctly expanded as single cells while the PDX.008.CL cells showed collective invasion, a feature of metastasis (bars 8 h = 300 µm, bars day 4 = 150 µm). (**F**) Equal numbers of KTC1 control cells and PDX.008.CL cells were plated in soft agar and grown for 2 weeks, then colonies counted. Data demonstrated a significant higher number of PDX.008.CL colonies in comparison to KTC1 and KTC1 Clone 2 colonies. RAC1 amplification per se did not promote contact independence. *n* = 6, **** = *p* < 0.0001. (**G**) The size of PDX.008.CL colonies grown in soft agar is significantly bigger than that of KTC1 colonies.

RAC1 plays a critical role in the formation of actin stress fibers and focal adhesion assembly through PAK1 activation [32]. Figure 6A shows a clear difference in morphology between the original KTC1 cells and the KTC1 sub-clones. In particular, the KTC1 clone 2 (harboring 4 RAC1 copies) exhibited a rounded cell shape and resembled most closely the PDX.008.CL cells due to their accumulation of vacuoles (compare with Figure 1C). To examine the effect of RAC1 alterations on the actin cytoskeleton organization, we stained F-actin using DyLight™ 488-conjugated phalloidin. As presented in Figure 6B, additional copy number of *RAC1* in KTC1 cells (Clone 2) is associated with a thick layer of cortical actin, which is compatible with a round cell morphology. The migratory behavior of different cell types correlates with their shape [33]; cells with a circular shape spread on a flat surface tend to be stationary, while cells with an elongated shape (rectangular, triangular or spindle-like) are more likely to be motile. To determine if this behavior is seen here and test the role of *RAC1* gene amplification in cell motility, we measured the rate of migration of the different KTC1 clones and PDX.008.CL cells in Boyden chambers. Figure 6C confirmed that the 2D motility of KTC1 cells expressing additional copies of *RAC1* as well as that of PDX.008.CL cells were reduced compared to KTC1 control cells, correlating with their round morphology. To test further the influence of *RAC1* amplification on growth rate and motility, we performed 3D spheroid cultures of the KTC1 clones without Matrigel. Figure 6D shows that additional copies of *RAC1* significantly increased the size of the spheroids over time in comparison with regular KTC1 spheroids, which readily broke apart after four days of culture. To test invasive properties, we also assessed the behavior of KTC1 and PDX.008.CL cells in 3D Matrigel cultures. Both KTC1 and PDX.008.CL plated as single cells first grew as aggregates during the first 8h but were then able to expand in 3D Matrigel (Figure 6E, Day 4). They showed different invasive behaviors, with PDX.008.CL cells showing collective rather than single cell invasion. Further, we quantified the growth of isogenic clones and PDX.008.CL single cells in soft agar assays. Figure 6F,G demonstrate that (1) PDX.008.CL cells are anchorage-independent, which is not induced by *RAC1* gene amplification in KTC1 Clone 2, and (2) PDX.008.CL cells formed clones that are significantly larger and more numerous that KTC1 cell clones. Altogether, these experiments indicate that *RAC1* gene amplification impairs 2D cell motility, possibly due to induction of a rounder cell shape, but significantly increases growth rate, and perhaps collective cell migration in a 3D environment. This behavior is shown in response to gene amplification in the presence or absence of the P34R mutation.

### 2.10. Proto-Oncogenes Located on Chromosome 7 Are Amplified and Their Expression Is Upregulated in the PDX.008.CL Cell Line

**Figure 7:**
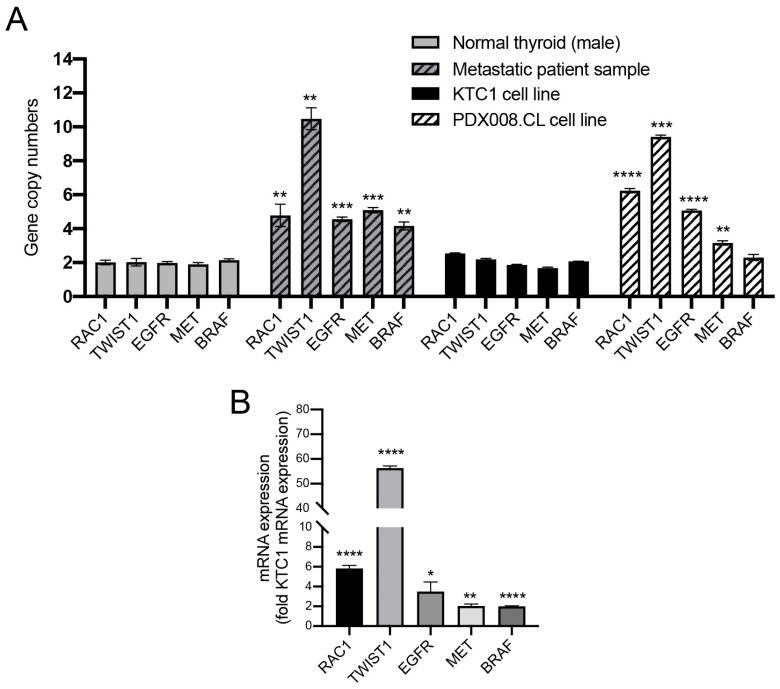
Copy numbers and expression of additional genes located on chromosome 7 in patient samples and PDX.008.CL cells. (**A**) There are additional copies of RAC1, TWIST1, EGFR1, MET and BRAF in comparison to normal thyroid, confirming aneuploidy of chromosome 7. High number of RAC1 and TWIST1 gene copies might indicate duplications/amplifications by chromothripsis at the tip of 7p. (**B**) High expression of genes located on chromosome 7 such as RAC1, TWIST1 and EGFR1 indicate a possible correlation between gene copy number and mRNA expression. *n* = 3–6, * = *p* < 0.05, ** = *p* < 0.005, *** = *p* < 0.0005, **** = *p* < 0.0001.

We next used the KTC1 and PDX.008.CL cell lines, as well as the metastatic patient sample to quantify copy number and expression of several cancer-related genes located on chromosome 7p and 7q, including *RAC1* (7p22.1) *TWIST1* (7p21.1), *EGFR1* (7p11.2), *MET* (7q31.2) and *BRAF* (7q.34). These genes are often over-expressed in a number of solid tumors, including thyroid cancer, and are linked to poor prognosis (Table S4) [34,35,36,37,38]. As seen in Figure 7A, the average *RAC1* copy number is 2 for KTC1 cells, 6 for PDX.008.CL cells, and 5 for the metastatic patient sample, as expected (see also Figure 5A). Moreover, additional copies of *TWIST1*, *EGFR1*, and *MET* were detected in both the patient sample and the PDX.008.CL cell line. Altogether these data indicate that both metastatic sample and derived cells harbor polyploidy of chromosome 7. mRNA expression of *RAC1*, *TWIST*, *EGFR1*, *MET* and even *BRAF* were also significantly higher in PDX.008.CL cells than in KTC1 cells. (Figure 7B). In particular, high expression of RAC1 and TWIST1 imply additional polyploidy events near the end of 7p in the PDX.008.CL cell line. These data suggest that copy number variations of *RAC1*, *TWIST1* and perhaps other genes located on chromosome 7 might be involved in acquired resistance to BRAF inhibitors in this patient.

## 3. Discussion

This study demonstrated that acquired *RAC1* gene alterations, more specifically amplifications and mutations, in a BRAF-mutated PTC patient metastasis induced changes in cell morphology, cell division, and growth in 3D in response to BRAF-inhibitor therapy. Dabrafenib resistance might be due to either or both the RAC1 (P34R) mutation and/or *RAC1* copy number amplification, as well as amplification of other genes located on polyploid chromosome 7. Pre-treatment tissue analysis of the original tumor showed an in-frame deletion of T599 (A598_T599delinsA) in the BRAF protein. A pre-treatment lymph node metastasis also demonstrated an acquired *BRAF* (K601N) (same allele) mutation. Both mutations are located in the kinase domain and increased the proliferation of mouse Ba/F3 hematopoietic cells, but not of the human non-malignant epithelial breast cells MCF10A. Although systematic studies have not been performed in thyroid cancers, studies in melanoma demonstrated that rare *BRAF* mutations located in the kinase domain of the protein (positions A598 to K601 in the protein) do respond well to kinase inhibitors such as vemurafenib and dabrafenib [11,39,40]. The patient presented at MD Anderson with neck and lung masses and was immediately treated with dabrafenib. He demonstrated a response in multiple neck nodes but progression in one prevascular mediastinal node. Because the mediastinal node was not in the radiation field during prior treatments, we assume that radiation did not influence the development of the mutation.

Understanding how cancer cells ultimately acquire resistance to targeted therapies remains challenging. In thyroid cancer, in particular BRAF-mutated PTC, activation of both intrinsic (RAF-MEK-ERK) and extrinsic (PI3K-AKT-mTOR) pathways have been proposed as mechanisms of resistance to BRAF inhibitors. These pathways can be activated through alternate BRAF splicing, c-MET overexpression, autocrine NRG1-mediated HER3 receptor activation, and *RAS* mutations [14,15,16,41,42,43,44,45]. More recently, our group has identified alternative BRAF activation of PAK1 to be important in the growth, invasion, and tumor forming effects of BRAFV600E in vitro and in vivo [27,28,29]. In addition to activation of alternate signaling pathways, genomic heterogeneity and instability of cancer cells under drug selection can lead to the emergence of more aggressive cell clones that have acquired secondary point mutations in driver genes [14,16], or gene amplifications [46]. Therefore, we initially hypothesized that the aggressive behavior displayed by the patient’s distant metastasis and derived cell line was due to secondary *RAC1* alterations.

RAC1 is a central small GTPase that is required for normal cell processes, including the cell cycle, cell–cell adhesion, cell motility, and differentiation. RAC1 is a canonical target of the integrin-FAK pathway and also of RAS and PI3 kinase. As such, activated RAC1 relays transforming information downstream of over-expressed FAK or mutated KRAS in cancer cells [47,48,49], mainly by binding to and activating its effectors PAK1-3 [50,51]. Interestingly, overexpression of wild-type RAC1 has been observed in many cancers [52,53,54,55,56] and correlates with poor prognosis [34] as well as resistance to chemotherapy [57,58]. PAK activity, a key result of RAC1 activation, has been demonstrated in the invasive fronts of aggressive PTCs, it forms a complex with BRAF in mitosis, and its activation is essential in BRAF-induced PTC tumorigenesis in mice [27,28]. Thus, an increase in RAC1/PAK activity by enhanced *RAC1* copy numbers and expression, even without a mutation, could explain our data showing a significant increase in spheroid sizes over time by KTC1 cells expressing additional wild-type RAC1 copies in comparison to control cells. However, the PDX.008.CL cells do not express more wild-type RAC1 protein than the control KTC1 cells, and therefore only over-expression of RAC1 (P34R) in these cells could account for resistance to dabrafenib. RAC1b, a hyperactive RAC1 splice variant, has also been detected in several tumor types [59,60,61] and seems to correlate with poor clinical outcomes when overexpressed in differentiated thyroid cancers [62,63]. However, the PDX.008.CL cells expressed this variant at very low levels corresponding to 2% of the total RAC1 transcripts (Figure S7). Hence, we infer that resistance of PDX.008.CL cells to dabrafenib is not linked to RAC1b overexpression. Recently, whole-exome sequencing of human melanoma samples revealed the existence of activating mutations affecting codons 28 and 29, such as RAC1 (F28L) and RAC1 (P29S) [21,64]. These mutations are found within the Switch I domain of the protein, and confer “fast-cycling” functional properties, in which GDP is exchanged more quickly for GTP [21]. RAC1 mutations such as P29S have clinically been associated with resistance to BRAF inhibitors in vitro and in vivo [23]. In an elegant study, Watson and colleagues demonstrated that the presence and overexpression of the RAC1 mutation in melanoma cell lines confer resistance to BRAF inhibitors and that silencing RAC1 restores sensitivity [18]. However, the functional difference between gene overexpression and mutation was not studied. Similarly, our data show that inhibiting RAC1 activity in PDX.008.CL cells by EHop-016 restores their sensitivity to dabrafenib. Because our patient tumor sample at progression and the PDX.008.CL cells showed also an increase in *RAC1* copy numbers, we chose to investigate the effects of *RAC1* amplification in the present study. An increase in *RAC1* wild-type copy numbers produced significant changes in the cellular morphology of KTC1 cells, generating a round shape similar to the PDX.008.CL cells and was associated with actin cytoskeleton reorganization. This change in morphology might favor cell growth and collective invasion, a feature of metastasis [65], rather than single cell migration. Altogether, our data indicated that resistance of PDX.008.CL cells to the kinase inhibitor might be due to an excess of RAC1 protein activity per se’ and that both increased RAC1 protein expression and the P34R mutation may be sufficient. The effect of a single copy of this mutation in isogenic PTC clones still needs to be determined. Excess RAC1 activity leads to an increase in phosphorylated PAK1 at Thr 423 and its activation, which promotes cell growth and might also lead to the abnormal organization of mitotic spindles [26,29].

We next sought to understand the cause(s) of the significant increase in expression of the RAC1 wild type and mutated protein in PDX.008.CL cells in comparison to other PTC cells. Flow cytometry data clearly established that the PDX.008.CL cell line is aneuploid. However, copy number assays and FISH analysis all pointed to polyploidy of chromosome 7, where *RAC1* is located. Our data indicate that *RAC1* copy number was diploid in the original patient sample, but significantly elevated in the metastatic sample. Therefore, trisomy of chromosome 7 and additional *RAC1* copy numbers might have been the cause of tumor progression. Although aneuploidy is found in ~90% of solid tumors [66], our understanding of its contribution to cancer initiation and progression is still limited. This is due to the fact that whole chromosome changes will alter the expression of many genes and that the consequences of copy number variations will be dependent on cell types. Identifying oncogenic drivers working through dosage changes is therefore challenging. Sack and colleagues generated a library of 30,000 barcoded human ORF to screen for genes that either stimulate or suppress proliferation when overexpressed in mammary, fibroblast, and pancreatic cells [67]. Many of these genes are not commonly mutated in cancer cells but were associated with copy number alterations and increased/decreased expression. Using their list of putative drivers, we selected genes on chromosome 7 that might be oncogenic if over-represented and over-expressed in thyroid cells, such as *TWIST1*, *EGFR*, *BRAF* and *MET*. We demonstrated amplification of *TWIST1*, *EGFR1* and *MET* genes in the mediastinal metastatic sample of the patient and the corresponding PDX.008.CL cells in comparison to KTC1. Expression levels of *RAC1* and *TWIST1* were significantly upregulated and mirrored their copy number. The expression of *BRAF* and *MET* was also elevated in comparison to KTC1 cells. Therefore, additional copies of these genes also may contribute to tumor progression and dedifferentiation that occurred under drug treatment. This is particularly true for the RAC1/PAK1 pathway, TWIST1 and EGFR1 proteins, which are known drivers of proliferation and EMT in aggressive thyroid cancer cells [27,29,35,43]. It is well known that benign thyroid lesions accumulate polysomies during their evolution from hyperplasia to goiter and adenomas [68], in particular trisomy 7. Trisomy 7 has also been observed in 66.0% of follicular and 70% of papillary thyroid cancers by in situ hybridization [69,70,71]. Both trisomies 7 and 12 have been observed in papillary carcinomas with a poor clinical outcome [72,73,74,75]. Interestingly, diffuse accumulation of mitochondria within Hürthle carcinoma cells is also associated with trisomy 7 [76]. Additionally, gain of chromosome 7 can also lead to genome-wide gene expression alterations and increases in interchromosomal contacts [77]. In addition to trisomy 7, we established the presence of further duplications of *RAC1* and amplification of *TWIST1* in the p22-p21 region that might be attributed to chromothripsis, which possibly arose during PDX development. It has been recently demonstrated that chromothripsis may lead to extrachromosomal circular DNA (ecDNA) amplification and re-integration preferentially near chromosome ends [78,79].

In conclusion, this study is the first to describe a *RAC1* (P34R) mutation and *RAC1* gene amplification as mechanisms of resistance and morphological dedifferentiation in response to *BRAF* inhibition in BRAF-mutated PTC. Our studies also suggest that polyploidy of chromosome 7 leading to increased expression of specific genes should be considered for aggressive thyroid tumor sample analysis and further treatments.

## 4. Materials and Methods

### 4.1. DNA Sequencing, Mapping, and Variant Calling

Three tumor samples were characterized by DNA sequencing. DNA was extracted and purified from two FFPE archival samples of tumor tissue obtained from the patient prior to dabrafenib treatment, using the Qiagen QIAamp DNA FFPE tissue kit (Qiagen, Germantown, MD, USA). The DNA was sequenced using the MD Anderson Cancer Center T200.1 target sequencing platform (Table S2). DNA was also isolated from one fresh frozen FNA sample collected from the patient at progression on dabrafenib treatment using the Wizard Genomic DNA Purification Kit (Promega, Madison, WI, USA) and sequenced using a whole-exome sequencing platform. The targeted (T200.1) library was generated using biotin-labeled probes (Roche NimbleGen) designed to capture all exons from 263 select genes. The whole-exome library was generated using the Illumina TruSeq Exome Enrichment Kit. The captured libraries were sequenced on a HiSeq 4000 (Illumina Inc., San Diego, CA, USA) with a version 3 TruSeq paired-end flowcell according to manufacturer’s instructions. The sequence data were mapped to the human reference genome hg19 using BWA software. Samtools and Bedtools were used to calculate the mapping rate and coverage for quality control (QC). Picard was used to remove duplicate reads and VarScan2 was used to make somatic variant calls. The called somatic variants were annotated using Variant Effect Predictor (VEP) and ANNOVAR. Unless otherwise specified, analyses were carried out using the statistical programming language R.

### 4.2. Somatic Variant Analysis

Somatic mutation allelic fractions were compared between the pre- and post-treatment samples. Only somatic mutations that were covered by both platforms were considered. Somatic mutations were prioritized by the number of PubMed search results including the gene name and ‘dabrafenib’ in the search terms along with the interaction network proximity to BRAF. Analyses were performed using customized functions in the statistical programming language R (packages: dplyr, readr, stringr, tidyr, igraph and RISmed). The presence of BRAF and RAC1 mutations were verified by manual inspection of mapped reads using Integrated Genomics Viewer (IGV).

### 4.3. Cell Line Establishment

The PDX.008.CL patient-derived cell line was established from a patient-derived xenograft model of the post-treatment sample. Solid tumor tissue was cut into 1 mm × 1 mm fragments. Liberase DH enzyme solution (Roche Diagnostics, Indianapolis, IN, USA) was added to a final volume of 10 mL per gram tissue. The tube was placed in a 37 °C shaker at moderate speed for 30 min. The cells were pelleted at 500× *g* at room temperature for 5 min. The supernatant was drawn and the cells were re-suspended in 5 mL of mouse fibroblast-conditioned medium with supplements [80,81]. The cell suspension was passed through a 100 µm cell strainer (Falcon, Thermo Fisher, Waltham, MA, USA) and transferred to a T25 flask (Falcon, Thermo Fisher, Waltham, MA, USA). The cells were maintained in the above conditioned medium in an incubator at 37 °C with 5% CO_2_ for 20 passages before considered established. STR fingerprinting was used to confirm their human origin and uniqueness (Figure S2). After establishment, the cell line was maintained in D-MEM/F12 (HyClone, G&E Healthcare Biosciences, Pittsburgh, PA, USA) supplemented with 1% Glutamax (Gibco, Thermo Fisher, Scientific, Waltham, MA, USA) and 10% FBS (Omega Scientific, Thermo Fisher Scientific, Waltham, MA, USA).

### 4.4. PDX.008.CL Cell Line RAC1(P34R) Sequencing

Cell line genomic DNA was isolated using the Wizard Genomic DNA Purification Kit (Promega) and RAC1 exon 2 was PCR amplified using sense strand primer (5′-TGCTAACACCGGGTACCTAAAC-3′) and antisense strand primer (5′-TCATCCAGTCTCTGTACCTCAC-3′). The PCR product was purified using High Pure PCR Product Purification Kit (Roche) and sequenced by conventional Sanger di-deoxynucleotide sequencing (Lone Star Labs, Houston, TX, USA) (Figure S1a). In addition, BRAF exon 15 was analyzed by NGS by the MGH CCIB DNA Core (Figure S1b).

### 4.5. Other Cell Lines

Additional cell lines used in this study included the human PTC cell line BCPAP (BRAF V600E hemizygous, DSMZ-Leibniz Institute German Collection of Microorganisms and Cell Cultures, Braunschweig, Germany), the human PTC cell line KTC1 (BRAF V600E heterozygous, generously provided by Dr. R. Schweppe, University of Colorado [82,83,84], the mouse pro B lymphocyte Ba/F3 cell line (Creative Bioarray, Shirley, NY, USA), and the human fibrocystic MCF10A cell line (ATCC, Manassas, VA, USA). KTC1 isogenic clones were also established that harbored additional copies of the RAC1 wild-type gene.

### 4.6. Stable Transduction Experiments

HEK293T cells, grown 4 days in media without antibiotics and at 50–70% confluency, were co-transfected using Lipofectamine 2000 (Thermo Fisher Scientific, Waltham, MA, USA) with the pMD2.G plasmid, the psPAX2 plasmid (both from Addgene, Watertown, MA, USA) and the pLOC RAC1 wild-type plasmid at a 1:3:4 ratio, respectively. Six hours later, media was changed to media with 1M HEPES, 0.85%NaCl (Lonza, Houston, TX, USA) added to a final concentration of 25 mM, pH 7.2–7.5. The supernatant was collected in 1 mL aliquots 24, 48, and 72 h later and frozen at −80 °C. For transductions, polybrene (EMD Millipore, Burlington, MA, USA) was added to media containing 25 mM HEPES final as above, and the media mixture added 1:1 to thawed virus. Then, the final mixture was added to ~70% confluent KTC1 cells following manufacturer’s instructions. One day later, the media mixture with the virus was removed and replaced with fresh culture media. Cells were expanded in D-MEM/F12 culture media containing 10% FBS and 30 µg/mL blasticidin.

### 4.7. Pharmacological Inhibitors

Both dabrafenib, a BRAFV600E inhibitor, and EHop-016, a RAC1 specific inhibitor, were purchased from Selleck Chemicals, Houston, TX, USA, and used at concentrations of 0–10 µM in 0.001% DMSO in cell cultures.

### 4.8. Conventional Western Blotting

Control cells (0.001% DMSO only) and cells treated with dabrafenib or EHop-016 were rinsed with PBS and lysed for 20 min at 4 °C with RIPA buffer supplemented with 3 cocktails of proteases and phosphatases inhibitors at 1:100 dilution (Sigma, St Louis, MO, USA, #P8340, #P0044, #P5726). Lysed cells were collected with a cell scraper, collected in microfuge tubes and passed 10 times through a syringe and 21-gauge needle. Samples were centrifuged at 2000× *g* and 4 °C, and supernatants were collected. Protein concentration was estimated using the Pierce Bradford Colorimetric Assay (BCA) (Thermo Fisher Scientific, Waltham, MA, USA) with absorbance at 562 nm measured with an Epoch 2 microplate spectrophotometer (BioTek, Winooski, VT, USA). Alternatively, protein concentration was measured using the NI (Non-Interfering) Protein Assay (G-Biosciences, St Louis, MO, USA). Samples were run on 4–12% sodium dodecyl sulfate (SDS)-polyacrylamide gels (Novex Bolt™, Thermo Fisher Scientific, Waltham, MA, USA) and transferred to 0.4 microns nitrocellulose membranes according to Novex Bolt™ standard protocols. Following transfer, the membranes were washed and blocked with 5% non-fat powdered milk in Tris-buffered saline (TBS) for 1 h at room temperature. The blots were incubated in primary antibody solutions (Table S3) overnight at 4 °C, then washed with 1× TBS-0.1% Tween and probed with appropriate secondary antibodies for 1 h at room temperature. The secondary antibodies used were LI-COR infrared fluorophore-conjugated secondary antibodies (1:2000, LI-COR Biosciences, Lincoln, NE, USA, Table S3) diluted in 5% powdered milk in TBS with 0.1% Tween 20. The blots were visualized using an Odyssey Fc Imaging system (LI-COR Biotechnology, Lincoln, NE, USA). Band intensities were quantified after normalization over TUBA1A (Tubulin) or VCL (Vinculin) using ImageJ (NIH). The band intensities were compared to controls for relative quantification. Each data point represents at least 2 technical replicates for each of 3 independent experiments.

### 4.9. Quantitative Western Blotting

Capillary-based Western blots were performed using a Wes automated system (ProteinSimple, San Jose, CA, USA). All protein lysates were harvested from cells near 75% confluency using a RIPA buffer, except lysates used for active PAK1 assays, which were harvested near 25% confluency. Protein lysates were mixed with a 5× sample buffer containing SDS, DTT, and internal fluorescent molecular weight standards, and heated at 95 °C for 5 min. Heated samples were loaded onto a plate containing capillary-specific wells loaded with protein standards, stacking and separation matrices, blocking and wash buffers, primary and HRP-conjugated secondary antibody solutions, and detection reagents (streptavidin-HRP and luminol-S/peroxide). Antibody dilutions were empirically determined. Data analyses of detected protein peaks were performed using the Compass software (ProteinSimple) on Wes™ in the high-dynamic range image setting. Target protein peaks were standardized to the protein loading control VCL (vinculin), detected concurrently within the same capillaries.

### 4.10. RAC1 Activity Assay

An active RAC1 detection kit (Cell Signaling Technology, Danvers, MA, USA) was used to detect GTP-bound RAC1. Cells were rinsed with ice-cold PBS and harvested under non-denaturing conditions with ice-cold 1× Lysis/Binding/Wash Buffer (supplied with assay) with 1 mM PMSF, then vortexed briefly and incubated on ice for 5 min. Lysates were centrifuged at 16,000× *g* at 4 °C for 15 min and the supernatant transferred to a new tube. An aliquot of each supernatant was saved and denatured with the addition of 1.0% NP-40 and 0.1% SDS for total protein load control assessment. In early experiments, aliquots of non-denatured lysates were treated with GTPγS (positive control) and GDP (negative control) per the manufacturer’s protocol, to ensure the immunoprecipitation procedures worked properly. For active RAC1 (GTP-bound) detection (endogenous and controls), nondenatured lysates were immunoprecipitated with a slurry containing GST-PAK1-PBD fusion protein and glutathione resin per the manufacturer’s protocol. Eluted samples were then probed for RAC1 in Western blots using a mouse monoclonal Ab provided with the assay (Cell Signaling Technology, Danvers, MA, USA, Table S3) and standardized using rabbit anti-vinculin with Wes technology as described above.

### 4.11. PAK1 Activity Assay

KTC1, KTC1 isogenic cells and PDX.008.CL cells were rinsed with PBS and treated for 20 min at 4 °C with NP40 lysis buffer supplemented with 3 cocktails of proteases and phosphatases inhibitors, 1:100 dilution (Sigma, St Louis, MO, USA, #P8340, #P0044, #P5726) and proteins extracted as described above. Intracellular PAK1 activity was determined in total lysate and by immunoprecipitation (IP) using a PAK1 antibody (Table S3) followed by Western blot detection of phospho-PAK1 at Thr423 (Table S3), which is required for activation of PAK1 [30,31].

### 4.12. RT-qPCR Analysis

Total RNA isolation was carried out using the Qiagen RNeasy kit (Qiagen, Germantown, MD, USA) and cDNA was obtained with MuLV reverse transcriptase (Thermo Fisher Scientific, Waltham, MA, USA). Quantitative real-time PCR (qRT-PCR) was performed using the TaqMan Gene Expression Mastermixes (Thermo Fisher Scientific, Waltham, MA, USA). TaqMan Gene Expression Assays are listed in Table S5. Relative quantitative fold change was determined using the ΔΔCt method. In all analyses, the expression value of each gene was normalized to the amount of internal control genes cDNA (*RPLPO* and *GAPDH)* to calculate a relative amount of RNA in each sample. Results obtained using different internal control genes were not significantly different. Quantitative relative fold changes in comparison to experimental controls were calculated. qRT-PCR was carried out with 6 technical replicates per sample and experiment, and at least three independent experiments. *p*-values ≤ 0.05 were denoted significant.

### 4.13. Transient Viability Assays

Lentiviral particles were made using the pHAGE vector expressing RAC1 (P29S), RAC1 (P34R), RAC1 WT, BRAF (K601N), and BRAF (A598_T599delinsA) [24], together with packaging plasmids, and used to infect Ba/F3 cells and MCF10A cells. Simultaneously, a set of negative controls (mCherry and luciferase genes only) were prepared. After infection, cells were cultured in media lacking essential growth factors (IL-3 for Ba/F3 and EGF and insulin for MCF10A). Since the parental Ba/F3 and MCF10A cells grow poorly without these specific growth factors in serum-free medium (Bollig-Fisher, PLOSOne, 2011), we expected that significant cell proliferation in their absence would be caused by driver mutations. Cell viability of the infected cells was measured by CellTiter-Glo (Promega) at 0.5, 1.0, 1.5, and 2 weeks post-infection.

### 4.14. Actin Staining

Cells were cultured in 35 mm tissue culture dishes for 48 h, then rinsed briefly with PBS containing 4% (*w*/*v*) paraformaldehyde, then fixed with 4.0% (*w*/*v*) paraformaldehyde diluted in PBS for 15 min at room temperature (RT). Samples were permeabilized with 0.3% Triton X-100 in TTBS (0.05% Tween 20 in 1× TBS (Santa Cruz Dallas, TX, USA) for 10 min, then washed 3 times 5 min each with TTBS at RT. Fixed cells were then exposed to DyLight™ 488-conjugated phalloidin (Cell Signaling Technology, Danvers, MA, USA) in TTBS containing 1.0% BSA for 1 h at RT, then counterstained with DAPI and imaged using a Nikon Eclipse Ti2 phase contrast/fluorescence microscope.

### 4.15. Cell Growth Assays

Equal numbers of cells (~500/well) were plated in octuplicate and grown in five 96-well plates containing DMEM/F12 supplemented with 10% serum. One plate was collected and fixed (4% PFA) prior to treatment (day 0) to be used to determine starting number of cells for each cell line or subpopulation. The wells in the remaining plates were either treated with DMSO or with individual or combined inhibitors (Dabrafenib and EHop-016) at concentrations of 0.001, 0.01, 0.1, 0.25, 0.5, 1.0, 1.75, 2.5, 5.0, 7.5, 10.0 μM. Cells were allowed to grow for 4 days following treatments and then stained with the nuclear dye DAPI just prior to entire well tile imaging using a high throughput IN Cell Analyzer (GE Healthcare Bio-Sciences, Pittsburgh, PA, USA) kindly made available by the High-Throughput Screening Core Lab, Institute for Biosciences and Technology, Texas A&M Health Science Center, Houston, TX, USA. Images were then stitched together and cells counted using the system’s image analysis software. Relative cell growth was calculated following normalization to the starting cell number (day 0 values) for each treatment group. In-house R code using logistic curves was used to estimate IC_50_ values with the delta method to calculate variance and two-sample t-tests were used to calculate significant differences between cell lines.

### 4.16. Migration and Invasion Assays

Each cell line was suspended in serum-free DMEM/F12 medium, with or without 1 μM dabrafenib. One hundred thousand cells were plated (100 μL volume/well) onto 8 µm-pore inserts (24-well plates) that were either tissue culture-treated (Corning Life Sciences, Lowell, MA, USA, #3422) or Matrigel^®^-coated (Corning Life Sciences, Lowell, MA, USA, #354480) for migration and invasion assays, respectively. The bottom chambers (receiving chamber) of the 24-well plates were filled with NIH 3T3 conditioned media. Cells that had migrated through the pores or invaded through Matrigel^®^-coated pores were stained with a Diff-Quick kit (Thermo Fisher Scientific, Waltham, MA, USA) and counted under the microscope.

### 4.17. Spheroid Formation, 3-D Growth, and Invasion

Cell lines were stably transduced with the non-toxic fluorescent nuclear dye, IncuCyte™ NucLight™ Red (Essen Bioscience, Ann Arbor, MI, USA), per manufacturers protocol. Equal numbers of cells (~1000) were plated and grown in round bottom ultra-low cell attachment 96-well plates (Corning Life Sciences, Lowell, MA, USA, #4515) containing 50 µL DMEM/F12 supplemented with 10% serum. Spheroid formation and growth were monitored in each well through hourly images over two days using the IncuCyte™ S3 SPHEROID scan (Essen Bioscience, Ann Arbor, MI, USA) at 10× magnification. Following the two-day incubation, spheroids were embedded in Matrigel diluted in culture media (1:1) and invasion into the matrix was monitored for an additional five days.

Additionally, KTC1, KTC1 Clone 1 and KTC1 Clone 2 lentivirus transduced cell lines were plated as above and IncuCyte pictures were taken on a 2 h cycle using the SPHEROID scan with phase contrast and brightfield options at 10× magnification. Plates were cultured and pictures taken for 4 days. Images were analyzed with the IncuCyte software.

### 4.18. Soft Agar Colony Formation Assay

KTC1 isogenic cell lines or PDX.008.CL cells (10,000 cells) were mixed with 1 mL of 0.36% Bactoagar solution in DMEM supplemented with 10% fetal bovine serum and layered on top of a 0.6% Bactoagar layer in DMEM. Plates were incubated at 37 °C in 5% CO_2_ for two weeks. Colonies were counted and photographed. The results were expressed as the average of counts from six plates.

### 4.19. Flow Cytometry

Cells were treated with 0.1 µM dabrafenib or DMSO (0.001%) in culture media for 48 h, then harvested and fixed in ice-cold 70% ethanol for 24 h, washed and rehydrated in PBS. DNA staining was achieved by treating the cells with RNAse A (10mg/mL) and with propidium iodide (10 mg/mL) (Sigma, St Louis, MO, USA) for 24 h and analyzed using a Gallios flow cytometer (Beckman Coulters, Brea, CA, USA).

### 4.20. Gene Copy Number Assay

Genomic DNA was extracted from cell lines and tumor samples using the Wizard Genomic DNA Purification Kit (Promega, Madison, WI, USA) according to the manufacturer’s instructions. DNA quality (260:280 ratio) and concentration were assessed by NanoDrop (ND-100 spectrophotometer, Thermo Fisher Scientific, Waltham, MA, USA) and then diluted to generate a uniform DNA concentration (5 ng/μL per replicate/reaction). Because the amount of patient tumor gDNA available was insufficient to test all genes, gDNA was also whole-genome amplified using the WAG2 GenomePlex Complete Whole Genome Amplification Kit (Sigma-Aldrich, St Louis, MO, USA). Quality control for accuracy was performed by comparing with data obtained with amplified gDNA from known tumor samples and cell lines, including PDX.008.CL. We used a TaqMan-based copy number assay specific for *RAC1* (NM_006908.4, Chr.7: 6374495-6403967, position 6388008, on build GRCh38) (assay ID# Hs04938353_cn, Thermo Fisher Scientific, Waltham, MA, USA). The copy number assay human RNAase-P (Life Technologies, #4403328, Carlsbad, CA, USA) was used as a reference. Briefly, each 20-μL reaction mixture was run in a 96-well plate with four replicates per sample, each containing five μL of DNA sample at five ng/mL, ten μL of TaqMan master, one μL of copy number assay for *RAC1*, and one μL of reference assay RNAase-P. The real-time polymerase chain reaction system’s program was 10 min of polymerase activation at 95 °C, followed by 40 cycles at 95 °C for 15 s and 60 °C for 1 min. The data were imported to CopyCaller Software V2.0 (Life Technologies) for comparative Ct (delta-delta CT) relative quantification analysis of the real-time data. The comparative CT (delta-delta CT) method first calculates the difference (delta CT) between the threshold cycles of the target and reference assay. Then, the method compares the delta CT values of the test samples to a calibrator sample that contains a known number of copies of the target sequence (human genomic DNA, male and female (Catalog # G1521, G1571, Promega, Madison, WI, USA). The analysis parameters for VIC- CT detection threshold were set to 32 cycles, and the calibrator sample copy number was set at two. The copy number calculated by the relative quantitation of (1.6 to 2.15) is considered two copies. Alternatively, to compare the CNV of different genes at once, the same procedure was adapted for 384-well plates run on a BioRAD CFX384 Real-Time system. TaqMan assays for additional gene copy numbers are listed in Table S5.

### 4.21. Fluorescence In Situ Hybridization (FISH)

For evaluation of RAC1 gene copy number status, PDX.008.CL and KTC1 control diploid cells were cultured in 10 cm Petri dishes to about 70% confluency and treated with colcemid (0.04 μg/mL) for 4 h. The cells were then transferred to 15 mL conical tubes and centrifuged at 400× *g* for 7 min. They were then subjected to hypotonic treatment (0.075 M KCl) for 20 min at room temperature and centrifuged. After resuspension, the cells were fixed in a methanol and acetic acid mixture (3:1 *v*/*v*) for 15 min, washed three times with the fixative, spread on slides, and air-dried. FISH was performed on the above cytological preparations using a *RAC1* FISH probe and a chromosome 7 centromeric probe (Empire Genomics, Buffalo, NY, USA) according to the manufacturer’s instructions with slight modifications. Briefly, 2 μL of each of the two probes were mixed with 6 μL of the in situ hybridization buffer. The mixture was applied to the slide, covered with a glass coverslip (22 × 22 mm), and sealed with rubber cement. The slides were then denatured at 72–73 °C using the Thermobrite system (Abbott Laboratories, IL, USA) and incubated at 37 °C overnight. They were washed using 2× SSC 45–70 °C for 1–2 min, counterstained with DAPI and analyzed using a Nikon 80i microscope and the Metaview Imaging Software (Molecular Devices, San Jose, CA, USA) using the green and orange fluorescence channels. Overlapping nuclei were excluded and individual and well-defined nuclei were analyzed. One hundred nuclei were scored and the number of red (gene-specific) and green (chromosome-specific) signals were recorded.

## 5. Conclusions

In conclusion, this study identified a role for *RAC1* gene amplification, as well as polyploidy of chromosome 7, as a mechanism of dedifferentiation of PTC into ATC, increased rate of cell proliferation and part of the process of acquired resistance to the BRAF-inhibitor dabrafenib.

## Figures and Tables

**Table 1:** Somatic mutations captured by T200.1 and WES platforms.

HGNC	Chr	Start (GRCh37/hg19)	Ref (cDNA)	Alt	Type	Allele Frequency			HGNC_AAS	Oncogenic Prediction Score (CScape)
						Pre_1 ST (FFPE) T200.1	Pre_2 LN (FFPE) T200.1	Post (M) FNA (WES)		
BRAF	7	140453191	TAC	-	DEL	17.7	33.7	18.2	BRAF_T599del	n/a
BRAF	7	140453132	A	T	SNV	0	35.6	24.7	BRAF_K601N	0.66
RAC1	7	6426908	C	G	SNV	0	0	25.6	RAC1_P34R	0.85
PIK3CA	3	18942499	G	T	SNV	0	6.6	0	PIK3CA_C769Fl	0.92
POS1	6	117687245	C	A	SNV	0	5.0	0	ROS1_L936M	0.71
BRCA1	17	41244936	C	T	SNV	0	8.1	0	BRCA1_P871L	0.43
XPO1	2	61713042	G	T	SNV	0	5.1	0	XPO1_R790I	0.95
NBN	8	90990479	G	C	SNV	7.0	9.7	0	NBN_E185Q	0.79
NBN	8	90958530	A	G	SNV	9.3	0	0	NBN_splice_region_variant	n/a
BRCA1	17	41223094	A	G	SNV	7.5	0	0	BRCA1_S1634G	0.74
BRCA1	17	41244000	A	G	SNV	7.9	0	0	BRCA1_K1183R	0.37
FLT3	13	28624294	C	T	SNV	6.3	0	0	FLT3_T227M	0.53
TSHR	1	81528564	G	T	SNV	8.3	0	0	TSHR_splice_region_variant	n/a
SETD2	3	47125385	C	T	SNV	12.8	0	0	SETD2_P1962L	0.46
ATR	3	142217564	C	A	SNV	4.9	0	0	ATR_A1811A	0.30
ATR	3	142274740	A	-	DEL	8.6	0	0	ATR_I774fs	n/a
MED12	X	70361760	A	-	DEL	4.6	0	0	MED12_Q2149fs	n/a
GABRA6	5	161114522	A	G	SNV	7.1	0	0	intron	n/a

ST = soft tissue lesion (pre-treatment) from FFPE DNA, LN = cervical lymph node (pre-treatment) from FFPE DNA, M = mediastinal lymph node (progression) from FNA DNA.

## Data Availability

The data presented in this study are available on request from the corresponding author.

## Supplementary Materials

The following are available online at https://www.mdpi.com/article/10.3390/cancers13194950/s1, Figure S1: A: Sanger sequencing of *RAC1* exon 2, after PCR amplification with primers framing codon 34. B: NGS analysis of *BRAF* exon 15. Figure S2: STR analysis of KTC1 and PDX.008.CL cells. Figure S3: Wes digital capillary protein analysis of RAC1 expression. Figure S4: Conventional Western blot analysis of RAC1 expression with different pharmacological inhibitors and antibodies. Figure S5: Wes digital capillary protein analysis of activated RAC1. Figure S6: Conventional Western blot analysis of PAK1 activation. Figure S7: Expression of *RAC1b* mRNA in comparison to *RAC1* in 2 PTC cell lines and the PDX.008.CL cells. Table S1: CM50 Gene Panel. Table S2: T200.1 Gene Panel. Table S3: Primary and secondary antibodies. Table S4: List of probed proto-oncogenes located on chromosome 7. Table S5: TaqMan and IDT probes/primers used for RT-qPCR and copy number assays.
