# Supplementary material for: RAC1 Alterations Induce Acquired Dabrafenib Resistance in Association with Anaplastic Transformation in a Papillary Thyroid Cancer Patient
Source: Cancers (Basel). 2021 Sep 30;13(19):4950. doi: 10.3390/cancers13194950 (PMC8507731; doi:10.3390/cancers13194950)
Supplement: Supplementary file 1 [file cancers-13-04950-s001.zip › cancers-1367794-supplementary.pdf]

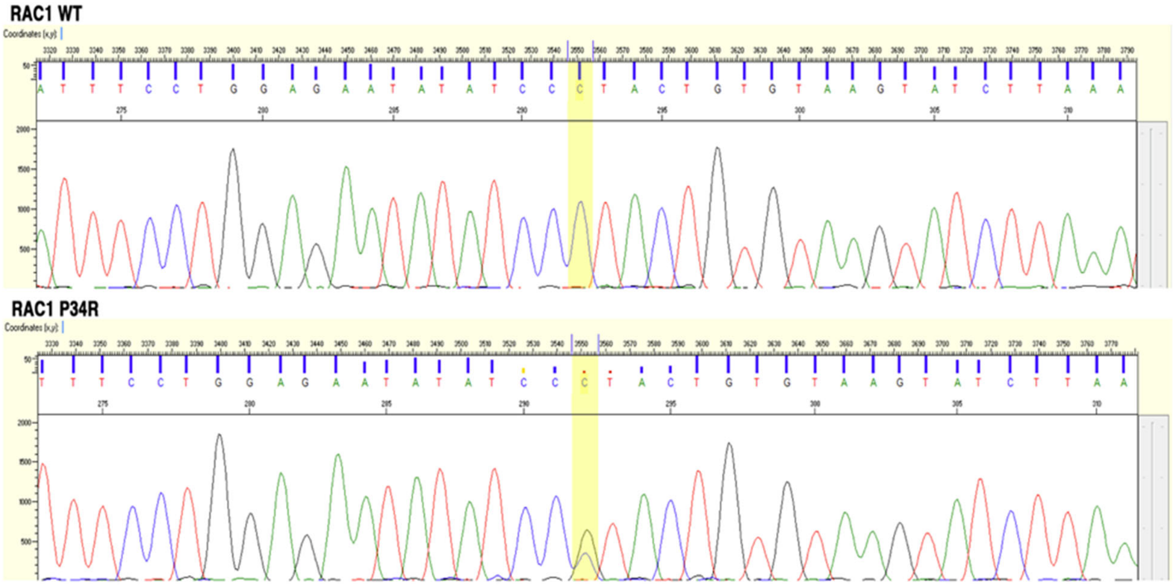


**A**

PDX.008.CL_NCBI_NG_007873.3_201
CTCTTCATAATGCTTGCTCTGATAGGAAAATGAGATCTACTGTTTTCCTTTACTTACTACACCTCAGATATATTTCTTCATGAAGACCTCACAGTAAAAATAGGT
GATTTTGGTCTAGCTACAGTGAAATCTCGATGGAGTGGGTCCCATCAGTTTGAACAGTTGTCTGGATCCATTTTGTGGATGGTAAGAATTGAGGCT
A T V K

> PDX.008.CL _NC12_CONTIG_198_p1 59467 pairs of NGS reads, 74.52%
CTCTTCATAATGCTTGCTCTGATAGGAAAATGAGATCTACTGTTTTCCTTTACTTactacacctcagatatatttcttcatgaagacctcacagtaaaaataggtgattttggtctagcag
tgaattctcgatggagtgggtccCATCAGTTTGAACAGTTGTCTGGATCCATTTTGTGGATGGTAAGAATTGAGGCT A
V N

> PDX.008.CL _NC12_CONTIG_201_p2 20325 pairs of NGS reads, 25.47%
CTCTTCATAATGCTTGCTCTGATAGGAAAATGAGATCTACTGTTTTCCTTTACTTACTacacctcagatatatttcttcatgaagacctcacagtaaaaataggtgattttggtctagct
acagtgaaatctcgatggagtggGTCCCATCAGTTTGAACAGTTGTCTGGATCCATTTTGTGGATGGTAAGAATTGAGGCT A
T V K

KTC_NCBI_NG_007873.3_201
CTCTTCATAATGCTTGCTCTGATAGGAAAATGAGATCTACTGTTTTCCTTTACTTACTACACCTCAGATATATTTCTTCATGAAGACCTCACAGTAAAAATAGGT
GATTTTGGTCTAGCTACAGTGAAATCTCGATGGAGTGGGTCCCATCAGTTTGAACAGTTGTCTGGATCCATTTTGTGGATGGTAAGAATTGAGGCT
A T V K

>KTC_NC11_CONTIG_201_p1 40686 pairs of NGS reads, 51.74%
CTCTTCATAATGCTTGCTCTGATAGGAAAATGAGATCTACTGTTTTCCTTTACTTACTacacctcagatatatttcttcatgaagacctcacagtaaaaataggtgattttggtctagct
acagtgaaatctcgatggagtggGTCCCATCAGTTTGAACAGTTGTCTGGATCCATTTTGTGGATGGTAAGAATTGAGGCT A
T V K

>KTC_NC11_CONTIG_201_p2 37941 pairs of NGS reads, 48.25%
CTCTTCATAATGCTTGCTCTGATAGGAAAATGAGATCTACTGTTTTCCTTTACTTACTacacctcagatatatttcttcatgaagacctcacagtaaaaataggtgattttggtctagct
acagagaaatctcgatggagtgggTCCCATCAGTTTGAACAGTTGTCTGGATCCATTTTGTGGATGGTAAGAATTGAGGCT A
T E K

= normal sequence ATVK

= V600E mutation (ATEK)

= deletion of Threonine (T) and K601N mutation (AVN). Comes from deletion of TAC, which produces GCA instead of GCT (still an Alanine) but deletes ACA (Threonine), plus point mutation at K601

**B**

**Figure S1. A:** Sanger sequencing of RAC1 exon 2, after PCR amplification with primers framing codon 34. **B:** NGS analysis of BRAF exon 15 (MGH CCIB DNA Core)

THE UNIVERSITY OF TEXAS MD Anderson Cancer Center Making Cancer History®

# CLC

## Cell Line Characterization

**Cytogenetics and Cell Authentication core**

**CCAC database is a comprehensive databases of short tandem repeat (STR) profiles, The database includes profiles from 20 public database/ publications and the cell lines developed by MD Anderson research labs.**
**The database contains over 4650 unique human cancer cell line STR profiles, one of the largest cancer cell line STR online search database in the world**

***For questions please contact Xuesong Li (713)-792-6833 , Xsli@mdanderson.org***

THE UNIVERSITY OF TEXAS MD Anderson Cancer Center Making Cancer History®

**Annual cell line authentication is required by**
**UTMDACC INSTITUTIONAL POLICY # ACA1044**

| SET 518 | 2/25/2020 |
|---|---|

Expiration date: 2/25/2021

| Source | Sample_Name | AMEL | CSF1PO | D13S317 | D16S539 | D18S51 | D21S11 | D3S1358 | D5S818 | D7S820 | D8S1179 | FGA | TH01 | TPOX | vWA | Comments |
|---|---|---|---|---|---|---|---|---|---|---|---|---|---|---|---|---|
| Hofmann Marie Claude | KTC1 | X,Y | 10,12 | 11,12 | 12 | 12,13 | 29 | 14,15 | 11,12 | 11 | 11,14 | 23,26 | 9 | 11 | 14,17 | |
| CCLC customer database -SET 262 | KTC1 | X,Y | 10,12 | 11,12 | 12 | 12,13 | 29 | 14,15 | 11,12 | 11 | 11,14 | 23,26 | 9 | 11 | 14,17 | MATCH |

| Source | Sample_Name | AMEL | CSF1PO | D13S317 | D16S539 | D18S51 | D21S11 | D3S1358 | D5S818 | D7S820 | D8S1179 | FGA | TH01 | TPOX | vWA | Comments |
|---|---|---|---|---|---|---|---|---|---|---|---|---|---|---|---|---|
| Hofmann Marie Claude | FNA001 | X,Y | 11,12 | 11,12 | 11 | 12,17 | 28 | 14,17 | 10,12 | 10,12 | 12,13 | 23,25 | 7,9.3 | 8 | 16 | Profile Unique<br>No match found in the database |

**Figure S2.** STR analysis of KTC1 and PDX.008.CL cells. FNA001 was the original name given to PDX.008.CL cells.

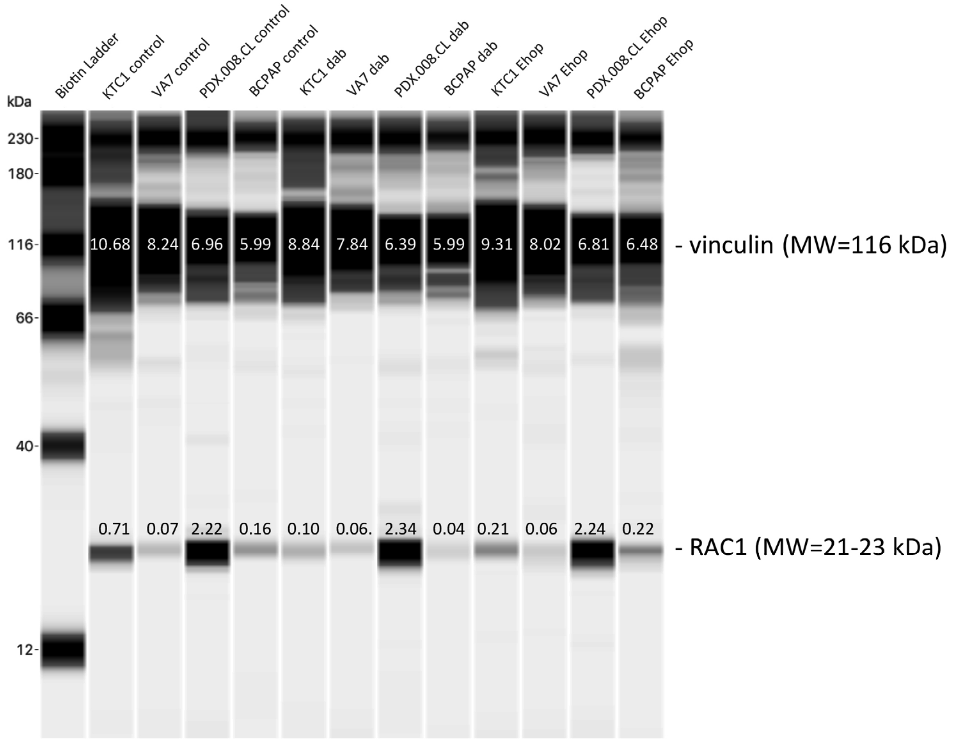


A

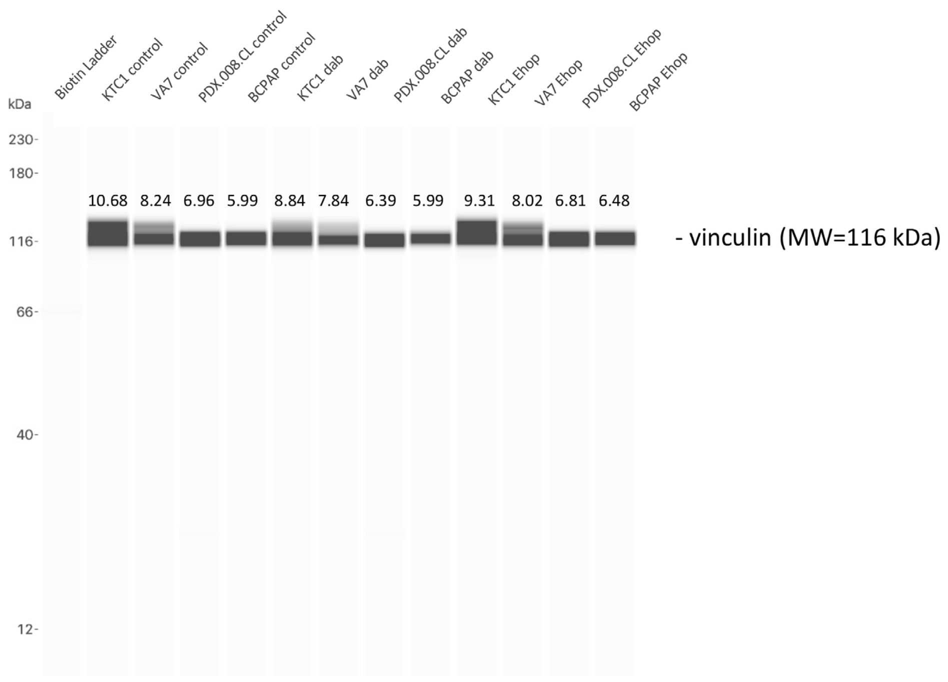


B

**Figure S3. A**: Wes digital capillary protein analysis of simultaneous total RAC1 and Vinculin expression in different papillary thyroid cancer cell lines treated with a BRAF V600E inhibitor (dabrafenib 0.1 μM) or RAC1 inhibitor (Ehop-016 0.1μM). Vinculin was used as loading control. **B**: Same capillary set, but brightness-adjusted for Vinculin. VA7 = KTC1 cell line with an acquired $KRAS^{G12D}$ mutation.

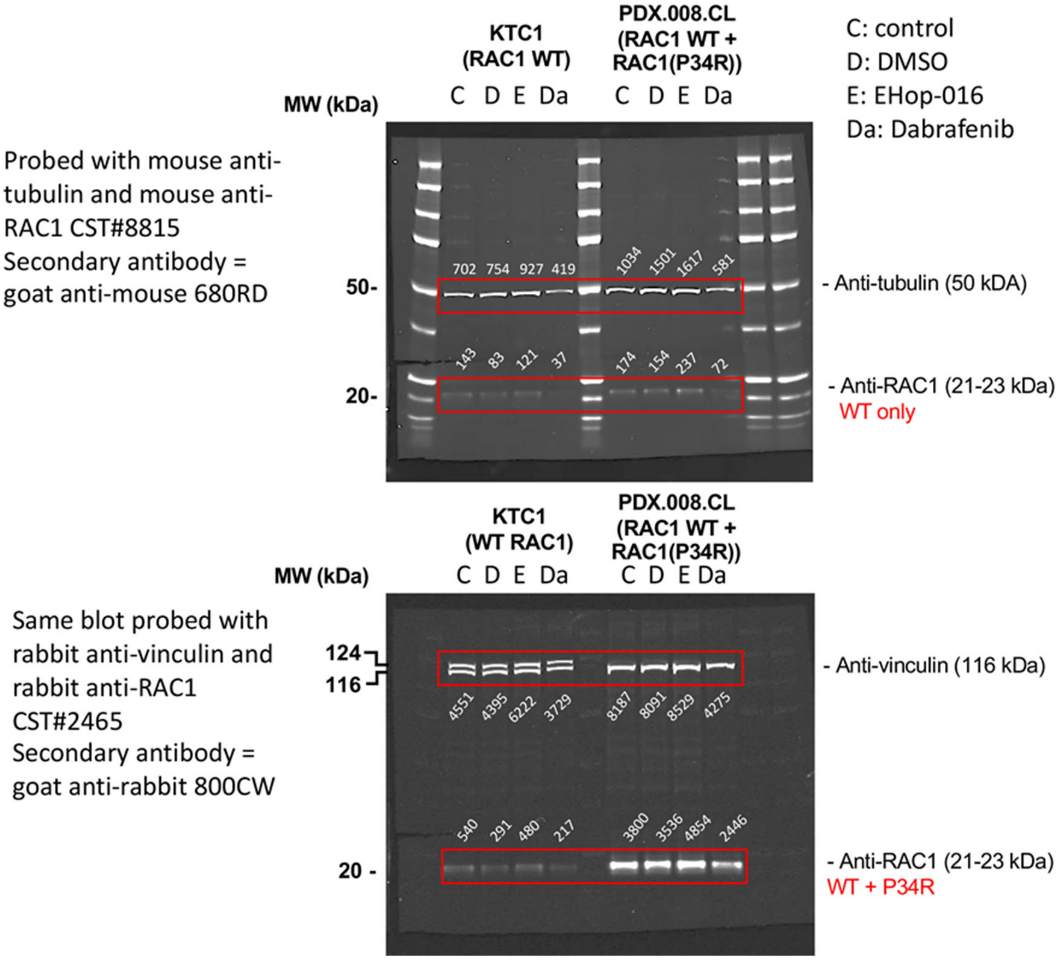


**A**

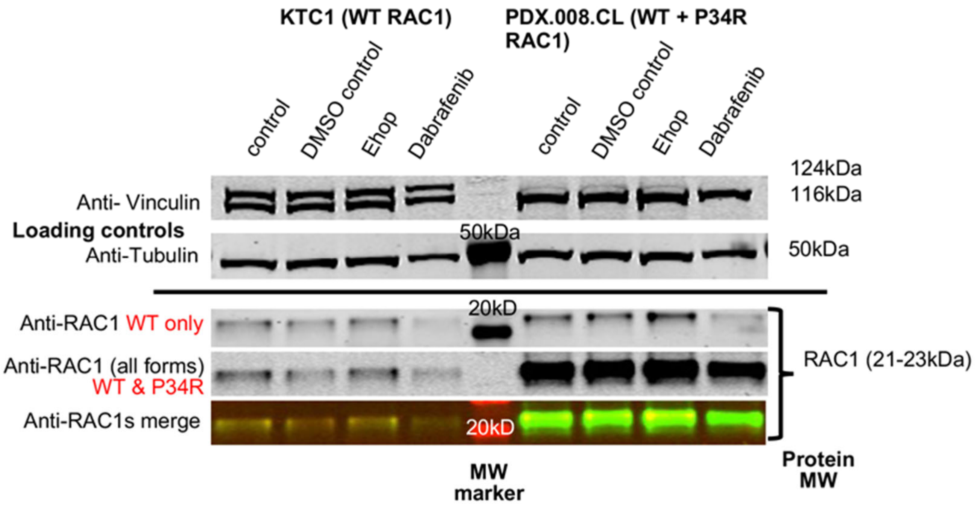


**B**

**Figure S4. A:** Conventional Western blot probing for RAC1, with vinculin or tubulin as simultaneous loading controls (LICOR fluorescence). KTC1 and PDX.008.CL were treated with and without the Ehop-016 RAC1 inhibitor or dabrafenib.

By subtracting non-P34R-detecting anti-RAC1 staining (detects only WT) from total anti-RAC1 staining (detects both WT and P34R forms), the relative amount of P34R mutant RAC1 can be calculated. **B:** Same blot, cut in strips and showing merged fluorescence for RAC1.

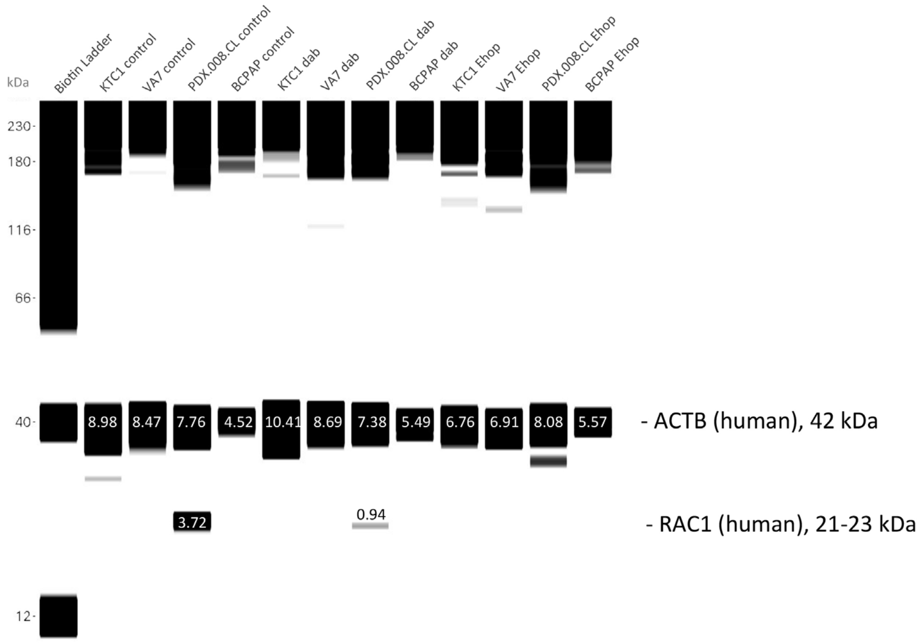


A

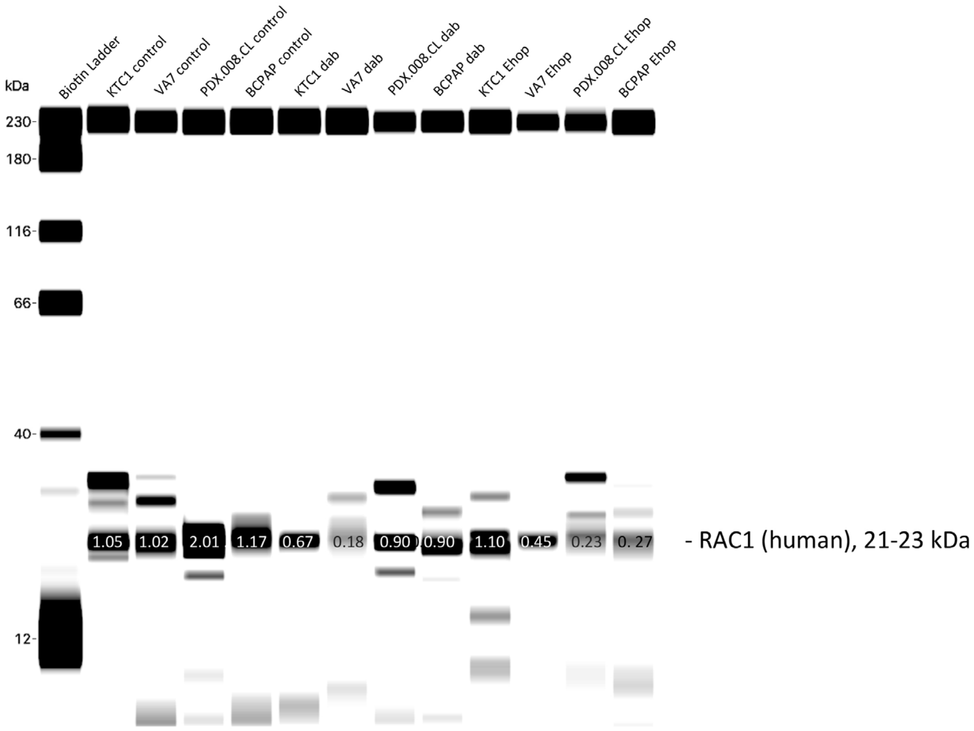


B

**Figure S5. A:** Wes digital capillary protein analysis of RAC1 and ACTB. ACTB is used as a loading control**. B:** Same samples after immunoprecipitation with a GST-PAK1-PBD fusion protein that strongly binds GTP-bound RAC1, then probed for total RAC1.VA7 = KTC1 cell line with an acquired KRAS$^{G12D}$ mutation.

Original blots for Figure 5D (chemiluminescence)

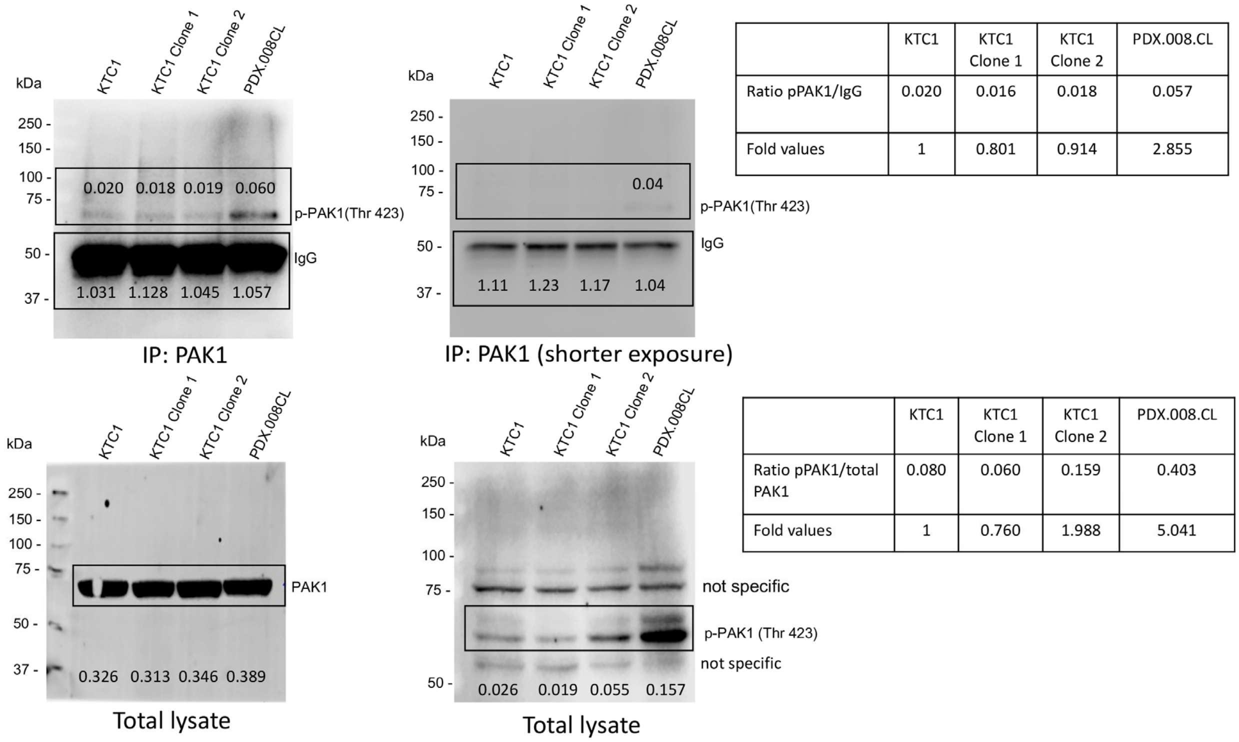


| | KTC1 | KTC1 Clone 1 | KTC1 Clone 2 | PDX.008.CL |
|---|---|---|---|---|
| Ratio pPAK1/IgG | 0.020 | 0.016 | 0.018 | 0.057 |
| Fold values | 1 | 0.801 | 0.914 | 2.855 |

| | KTC1 | KTC1 Clone 1 | KTC1 Clone 2 | PDX.008.CL |
|---|---|---|---|---|
| Ratio pPAK1/total PAK1 | 0.080 | 0.060 | 0.159 | 0.403 |
| Fold values | 1 | 0.760 | 1.988 | 5.041 |

**Figure S6.** Western blots of samples after immunoprecipitation with an anti-PAK1 antibody, then probing with a p-PAK (Thyr 423) antibody. PAK1 and pPAK1 in total lysates are also shown.

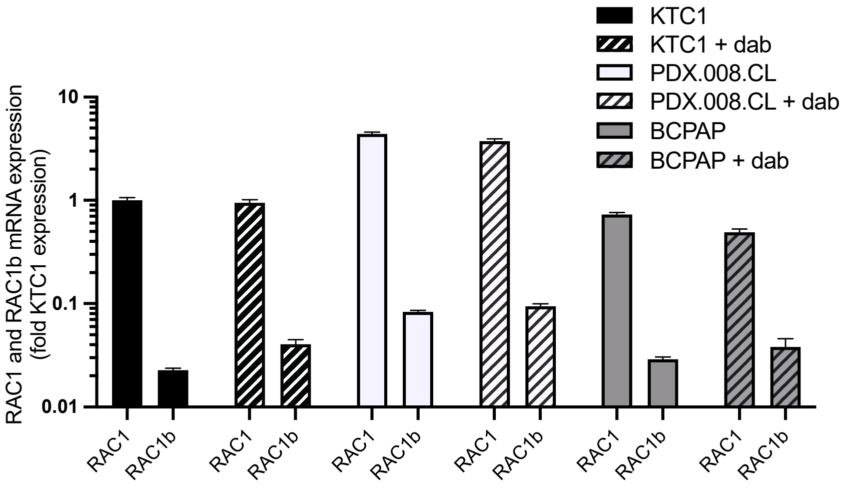


**Figure S7.** Expression of *RAC1b* mRNA in comparison to *RAC1* mRNA in two PTC cell lines and PDX.008.CL. *RAC1b* expression corresponds to 2% of total *RAC1* expression.

**Table S1.** CM50 Gene Panel.

| Gene /HGNC | Genomic Location | Strand | Accession Number | Exons (Codons) Tested | Gene Name |
|---|---|---|---|---|---|
| ABL1 | chr9:133589268-133763062 | + | NM_005157 | 4 (232-260), 5 (275-279), 6 (314-360), 7 (380-412) | ABL proto-oncogene 1, non-receptor tyrosine kinase |
| AKT1 | chr14:105235687-105262080 | - | NM_005163 | 3 (16-52), 6 (154-183) | AKT serine/threonine kinase 1 |
| ALK | chr2:29415640-30144477 | - | NM_004304 | 23 (1172-1204), 25 (1270-1279) | anaplastic lymphoma receptor tyrosine kinase |
| APC | chr5:112043202-112181936 | + | NM_000038 | 16 (860-891), 16 (1089-1125), 16 (1284-1326), 16 (1342-1384), 16 (1426-1471), 16 (1483-1524), 16 (1543-1582) | APC, WNT signaling pathway regulator |
| ATM | chr11:108093559-108239826 | + | NM_000051 | 8 (326-355), 9 (407-412), 12 (601-626), 17 (834-865), 26 (1292-1325), 34 (1674-1707), 35 (1726-1757), 36 (1790-1815), 39 (1926-1946), 50 (2436-2454), 54 (2650-2667), 55 (2682-2711), 56 (2718-2736), 59 (2865-2891), 61 (2933-2950), 63 (2996-3026), 63 (3041-3057) | ATM serine/threonine kinase |
| BRAF | chr7:140433813-140624564 | - | NM_004333 | 11 (439-473), 15 (581-611) | B-Raf proto-oncogene, serine/threonine kinase |
| CDH1 | chr16:68771195-68869444 | + | NM_004360 | 3 (65-96), 8 (337-374), 9 (380-408) | cadherin 1 |
| CDKN2A | chr9:21967751-21994490 | - | NM_000077 | 2 (51-90), 2 (98-140) | cyclin dependent kinase inhibitor 2A |
| CSF1R | chr5:149432854-149492935 | - | NM_005211 | 7 (297-319), 22 (953-973) | colony stimulating factor 1 receptor |
| CTNNB1 | chr3:41240942-41281939 | + | NM_001904 | 3 (9-48) | catenin beta 1 |
| EGFR | chr7:55086725-55275031 | + | NM_005228 | 3 (96-123), 7 (279-297), 15 (575-601), 18 (695-726),19-20 (729-796), 20 (807-823), 21 (855-875) | epidermal growth factor receptor |
| ERBB2 | chr17:37844393-37884915 | + | NM_004448 | 19-20 (752-797), 21 (839-882) | erb-b2 receptor tyrosine kinase 2 |
| ERBB4 | chr2:212240442-213403352 | - | NM_005235 | 3 (136-141), 4 (167-186), 6 (225-247), 7 (254-290), 8 (295-323), 9 (333-367), 15 (580-623), 23 (919-948) | erb-b2 receptor tyrosine kinase 4 |
| EZH2 | chr7:148504464-148581441 | - | NM_004456 | 16 (625-649) | enhancer of zeste 2 polycomb repressive complex 2 subunit |
| FBXW7 | chr4:153242410-153456172 | - | NM_033632 | 5 (264-287), 8 (378-403), 9 (434-473), 10 (478-509), 11 (567-594) | F-box and WD repeat domain containing 7 |
| FGFR1 | chr8:38268656-38326352 | - | NM_015850 | 4 (120-148), 7 (247-273) | fibroblast growth factor receptor 1 |
| FGFR2 | chr10:123237844-123357972 | - | NM_000141 | 7 (250-275), 7 (296-313), 9 9362-399), 12 (546-558) | fibroblast growth factor receptor 2 |

| | | | | | |
|---|---|---|---|---|---|
| FGFR3 | chr4:1795039-1810599 | + | NM_000142 | 7 (247-277), 9 (367-402), 14 (631-653), 16 (690-719), 18 (771-807) | fibroblast growth factor receptor 3 |
| FLT3 | chr13:28874483-29069265 | - | NM_004119 | 11 (437-466), 14 (570-610), 16 (663-685), 20 (828-847) | fms related tyrosine kinase 3 |
| GNA11 | chr19:3094408-3121454 | + | NM_002067 | 5 (202-219) | G protein subunit alpha 11 |
| GNAQ | chr9:80335191-80646219 | - | NM_002072 | 5 (206-245) | G protein subunit alpha q |
| GNAS | chr20:57414795-57486250 | + | NM_000516 | 8-9 (196-240) | GNAS complex locus |
| HNF1A | chr12:121416549-121440314 | + | NM_000545 | 3 (192-221), 4 (253-282) | HNF1 homeobox A |
| HRAS | chr11:532242-535550 | - | NM_005343 | 2 (5-35), 3 (42-82) | HRas proto-oncogene, GTPase |
| IDH1 | chr2:209100953-209119806 | - | NM_005896 | 4 (101-135) | isocitrate dehydrogenase (NADP(+)) 1, cytosolic |
| IDH2 | chr15:90627212-90645708 | - | NM_002168 | 4 (133-177) | isocitrate dehydrogenase (NADP(+)) 2, mitochondrial |
| JAK2 | chr9:4985245-5128183 | + | NM_004972 | 14 (603-622) | Janus kinase 2 |
| JAK3 | chr19:17935593-17958841 | - | NM_000215 | 4 (128-140), 13 (568-580), 16 (709-733) | Janus kinase 3 |
| KDR | chr4:55944426-55991762 | - | NM_002253 | 6-7 (244-291), 11 (471-480), 19 (872-894), 21 (961-988), 26 (1135-1156), 27 (1192-1221), 30 (1283-1310), 30 (1324-1357) | kinase insert domain receptor |
| KIT | chr4:55524095-55606881 | + | NM_000222 | 2 (23-58), 9 (494-514), 10-11 (525-587), 13 (627-661), 14 (664-684), 15 (714-724), 17 (802-828), 18 (832-858) | KIT proto-oncogene receptor tyrosine kinase |
| KRAS | chr12:25358180-25403854 | - | NM_004985 | 2-3 (5-66), 4 (114-150) | KRAS proto-oncogene, GTPase |
| MET | chr7:116312459-116438440 | + | NM_001127500 | 2 (159-188), 1 (339-378), 11 (816-856), 14 (981-1012), 16 (1105-1132), 19 (1246-1274) | MET proto-oncogene, receptor tyrosine kinase |
| MLH1 | chr3:37034841-37092337 | + | NM_000249 | 12 (373-415) | mutL homolog 1 |
| MPL | chr1:43803475-43820135 | + | NM_005373 | 10 (501-522) | MPL proto-oncogene, thrombopoietin receptor |
| NOTCH1 | chr9:139388896-139440238 | - | NM_017617 | 26 (1566-1602, 27 (1673-1680), 34 (2436-2476) | notch receptor 1 |
| NPM1 | chr5:170814708-170837888 | + | NM_002520 | 11 (283-295) | nucleophosmin (nucleolar phosphoprotein B23, numatrin) |
| NRAS | chr1:115247085-115259515 | - | NM_002524 | 2 (3-31), 3 (43-69), 4 (124-150) | neuroblastoma RAS viral oncogene homolog |
| PDGFRA | chr4:55095264-55164412 | + | NM_006206 | 12 (552-583), 14 (644-668), 15 (671-709), 18 (819-854) | platelet derived growth factor receptor alpha |
| PIK3CA | chr3:178866311-178952497 | + | NM_006218 | 2 (54-90), 2 (106-118), 5 (316-351), 7-8 (390-422), 8 (449-468), | phosphatidylinositol-4,5-bisphosphate 3- |

| | | | | | |
|---|---|---|---|---|---|
| | | | | 10 (522-549), 14 (677-720), 19 (898-924), 21 (1017-1051), 21 (1065-1069) | kinase catalytic subunit alpha |
| PTEN | chr10:89623195-89728532 | + | NM_000314 | 1 (1-25), 3 (55-70), 5 (99-135), 6 (165-184), 7 (212-215), 7 (231-267), 8 (282-300), 8 (312-342) | phosphatase and tensin homolog |
| PTPN11 | chr12:112856536-112947717 | + | NM_002834 | 3 (46-82), 13 (485-527) | protein tyrosine phosphatase, non-receptor type 11 |
| RB1 | chr13:48877883-49056026 | + | NM_000321 | 4 (130-159), 6 (196-203), 10 (314-345), 11 (350-366), 14 (452-463), 17-18 (547-582), 20 (655-691), 21 (703-724), 22 (743-770) | RB transcriptional corepressor 1 |
| RET | chr10:43572517-43625797 | + | NM_020975 | 10-11 (608-654), 13 (762-786), 15-16 (875-924) | ret proto-oncogene |
| SMAD4 | chr18:48556583-48611411 | + | NM_005359 | 3 (98-136), 4 (142-146), 5 (165-202), 6 (242-263), 8 (307-319), 9 (326-365), 10 (384-424), 1 (443-474), 12 (494-532) | SMAD family member 4 |
| SMARCB1 | chr22:24129150-24176705 | + | NM_003073 | 2 (35-72), 4-5 (144-206), 9 (373-386) | SWI/SNF related, matrix associated, actin dependent regulator of chromatin, subfamily b, member 1 |
| SMO | chr7:128828713-128853385 | + | NM_005631 | 3 (186-228), 5 (307-331), 6 (391-419), 9 (511-542), 11 (608-646) | smoothened, frizzled class receptor |
| SRC | chr20:35973088-36033821 | + | NM_005417 | 14 (499-533) | SRC proto-oncogene, non-receptor tyrosine kinase |
| STK11 | chr19:1205798-1228434 | + | NM_000455 | 1 (22-64), 4 (155-181), 4-5 (191-207), 6 (253-285), 8 (317-361) | serine/threonine kinase 11 |
| TP53 | chr17:7571720-7590868 | - | NM_000546 | 2 (1-20), 4 (68-113), 5 (126-138), 5-6 (149-223), 7 (225-258), 8 (263-307), 10 (332-367) | tumor protein p53 |
| VHL | chr3:10183319-10195354 | + | NM_000551 | 1 (78-108), 2 (114-150), 3 (155-174) | von Hippel-Lindau tumor suppressor |

**Table S2.** T200.1 Gene Panel.

| Gene | Genomic_Location | Strand | Accession Number (mRNA) | Name |
|---|---|---|---|---|
| ABL1 | chr9:133589268-133763062 | + | NM_005157 | ABL proto-oncogene 1, non-receptor tyrosine kinase |
| ABL2 | chr1:179068462-179198819 | - | NM_007314 | ABL proto-oncogene 2, non-receptor tyrosine kinase |
| ACVR1B | chr12:52345451-52390863 | + | NM_004302 | activin A receptor type 1B |
| ACVR2A | chr2:148602570-148688393 | + | NM_001278579 | activin A receptor type 2A |
| AJUBA | chr14:23440410-23451848 | - | NM_198086 | ajuba LIM protein |
| AKT1 | chr14:105235687-105262080 | - | NM_005163 | AKT serine/threonine kinase 1 |
| AKT2 | chr19:40736224-40791302 | - | NM_005163 | AKT serine/threonine kinase 2 |
| AKT3 | chr1:243651535-244006886 | - | NM_005465 | AKT serine/threonine kinase 3 |
| AKTIP | chr16:53525192-53537170 | - | NM_001012398 | AKT interacting protein |
| ALK | chr2:29415640-30144477 | - | NM_004304 | anaplastic lymphoma receptor tyrosine kinase |
| APC | chr5:112043202-112181936 | + | NM_000038 | APC, WNT signaling pathway regulator |
| AR | chrX:66763874-66950461 | + | NM_000044 | androgen receptor |
| ARAF | chrX:47420499-47431320 | + | NM_001654 | A-Raf proto-oncogene, serine/threonine kinase |
| ARID1A | chr1:27022522-27108601 | + | NM_006015 | AT-rich interaction domain 1A |
| ARID1B | chr6:157099064-157531913 | + | NM_017519 | AT-rich interaction domain 1B |
| ARID2 | chr12:46123620-46301819 | + | NM_152641 | AT-rich interaction domain 2 |
| ASXL1 | chr20:30946147-31027122 | + | NM_015338 | additional sex combs like 1, transcriptional regulator |
| ATM | chr11:108093559-108239826 | + | NM_000051 | ATM serine/threonine kinase |
| ATR | chr3:142168077-142297668 | - | NM_001184 | ATR serine/threonine kinase |
| ATRX | chrX:76760356-77041719 | - | NM_000489 | ATRX, chromatin remodeler |
| AURKA | chr20:54944445-54967351 | - | NM_198433 | aurora kinase A |
| AURKB | chr17:8108049-8113883 | - | NM_004217 | aurora kinase B |
| AXIN1 | chr16:337440-402676 | - | NM_003502 | axin 1 |
| AXIN2 | chr17:63524683-63557740 | - | NM_004655 | axin 2 |
| AXL | chr19:41725108-41767671 | + | NM_021913 | AXL receptor tyrosine kinase |
| B2M | chr15:45003685-45010357 | + | NM_004048 | beta-2-microglobulin |
| BAP1 | chr3:52435025-52444009 | - | NM_004656 | BRCA1 associated protein 1 |
| BCL11A | chr2:60678302-60780633 | - | NM_022893 | B-cell CLL/lymphoma 11A |

| | | | | |
|---|---|---|---|---|
| BCL2 | chr18:60790579-60986613 | - | NM_000633 | BCL2, apoptosis regulator |
| BCOR | chrX:39910499-40036582 | - | NM_017745 | BCL6 corepressor |
| BIRC2 | chr11:102217966-102249401 | + | NM_001166 | baculoviral IAP repeat containing 2 |
| BRAF | chr7:140433813-140624564 | - | NM_004333 | B-Raf proto-oncogene, serine/threonine kinase |
| BRCA1 | chr17:41196312-41277468 | - | NM_007294 | BRCA1, DNA repair associated |
| BRCA2 | chr13:32889617-32973809 | + | NM_000059 | BRCA2, DNA repair associated |
| BTK | chrX:100604435-100641212 | - | NM_000061 | Bruton tyrosine kinase |
| CARD11 | chr7:2945710-3083509 | - | NM_032415 | caspase recruitment domain family member 11 |
| CASP8 | chr2:202098166-202152434 | + | NM_001228 | caspase 8 |
| CBL | chr11:119076990-119178859 | + | NM_005188 | Cbl proto-oncogene |
| CCND1 | chr11:69455873-69469242 | + | NM_053056 | cyclin D1 |
| CCND2 | chr12:4382902-4414522 | + | NM_001759 | cyclin D2 |
| CCND3 | chr6:41902671-42016610 | - | NM_001136017 | cyclin D3 |
| CCNE1 | chr19:30302901-30315215 | + | NM_001238 | cyclin E1 |
| CD274 | chr9:5450503-5470567 | + | NM_014143 | CD274 molecule |
| CD79A | chr19:42381190-42385439 | + | NM_001783 | CD79a molecule |
| CD79B | chr17:62006098-62009704 | - | NM_000626 | CD79b molecule |
| CDC27 | chr17:45195311-45266665 | - | NM_001114091 | cell division cycle 27 |
| CDC73 | chr1:193091088-193223942 | + | NM_024529 | cell division cycle 73 |
| CDH1 | chr16:68771195-68869444 | + | NM_004360 | cadherin 1 |
| CDK12 | chr17:37617739-37690800 | + | NM_016507 | cyclin dependent kinase 12 |
| CDK4 | chr12:58141510-58146230 | - | NM_000075 | cyclin dependent kinase 4 |
| CDK6 | chr7:92234235-92465941 | - | NM_001259 | cyclin dependent kinase 6 |
| CDKN1B | chr12:12870302-12875305 | + | NM_004064 | cyclin dependent kinase inhibitor 1B |
| CDKN2A | chr9:21967751-21994490 | - | NM_000077 | cyclin dependent kinase inhibitor 2A |
| CDKN2C | chr1:51434367-51440309 | + | NM_001262 | cyclin dependent kinase inhibitor 2C |
| CEBPA | chr19:33790840-33793430 | - | NM_004364 | CCAAT/enhancer binding protein alpha |
| CHEK1 | chr11:125495031-125546150 | + | NM_001274 | checkpoint kinase 1 |
| CHEK2 | chr22:29083731-29137822 | - | NM_007194 | checkpoint kinase 2 |
| CIC | chr19:42788817-42799949 | + | NM_015125 | capicua transcriptional repressor |
| COL2A1 | chr12:48366748-48398285 | - | NM_001844 | collagen type II alpha 1 chain |
| CREBBP | chr16:3775056-3930121 | - | NM_004380 | CREB binding protein |
| CSF1R | chr5:149432854-149492935 | - | NM_005211 | colony stimulating factor 1 receptor |
| CTCF | chr16:67596310-67673088 | + | NM_006565 | CCCTC-binding factor |
| CTLA4 | chr2:204732511-204738683 | + | NM_005214 | cytotoxic T-lymphocyte associated protein 4 |
| CTNNB1 | chr3:41240942-41281939 | + | NM_001904 | catenin beta 1 |

| | | | | |
|---|---|---|---|---|
| CYLD | chr16:50775961-50835846 | + | NM_015247 | CYLD lysine 63 deubiquitinase |
| CYP2C19 | chr10:96522463-96612671 | + | NM_000769 | cytochrome P450 family 2 subfamily C member 19 |
| DAXX | chr6:33286335-33290793 | - | NM_001350 | death-domain associated protein |
| DDR2 | chr1:162602228-162750247 | + | NM_006182 | discoidin domain receptor tyrosine kinase 2 |
| DDX3X | chrX:41192651-41209524 | + | NM_001356 | DEAD-box helicase 3, X-linked |
| DICER1 | chr14:95552565-95623759 | - | NM_177438 | dicer 1, ribonuclease III |
| DNMT3A | chr2:25455830-25565459 | - | NM_175629 | DNA methyltransferase 3 alpha |
| EGFR | chr7:55086725-55275031 | + | NM_005228 | epidermal growth factor receptor |
| ELF3 | chr1:201979690-201986315 | + | NM_004433 | E74 like ETS transcription factor 3 |
| EP300 | chr22:41488614-41576081 | + | NM_001429 | E1A binding protein p300 |
| EPCAM | chr2:47596287-47614167 | + | NM_002354 | epithelial cell adhesion molecule |
| EPHA2 | chr1:16450832-16482582 | - | NM_004431 | EPH receptor A2 |
| EPHA3 | chr3:89156674-89531284 | + | NM_005233 | EPH receptor A3 |
| EPHA5 | chr4:66185281-66535653 | - | NM_004439 | EPH receptor A5 |
| ERBB2 | chr17:37844393-37884915 | + | NM_004448 | erb-b2 receptor tyrosine kinase 2 |
| ERBB3 | chr12:56473809-56497291 | + | NM_001982 | erb-b2 receptor tyrosine kinase 3 |
| ERBB4 | chr2:212240442-213403352 | - | NM_005235 | erb-b2 receptor tyrosine kinase 4 |
| ERCC2 | chr19:45854649-45873845 | - | NM_000400 | ERCC excision repair 2, TFIIH core complex helicase subunit |
| ERCC3 | chr2:128014866-128051752 | - | NM_000122 | ERCC excision repair 3, TFIIH core complex helicase subunit |
| ERCC4 | chr16:14014014-14046205 | + | NM_005236 | ERCC excision repair 4, endonuclease catalytic subunit |
| ERCC5 | chr13:103498191-103528351 | + | NM_000123 | ERCC excision repair 5, endonuclease |
| ESR1 | chr6:152011631-152424408 | + | NM_000125 | estrogen receptor 1 |
| ETV1 | chr7:13930856-14031050 | - | NM_004956 | ETS variant 1 |
| EZH2 | chr7:148504464-148581441 | - | NM_004456 | enhancer of zeste 2 polycomb repressive complex 2 subunit |
| FADD | chr11:70049269-70053508 | + | NM_003824 | Fas associated via death domain |
| FAM123B | chrX:63404997-63425624 | - | NM_139285 | APC membrane recruitment protein 1 (AMER1, WTX) |
| FANCA | chr16:89803959-89883065 | - | NM_000135 | Fanconi anemia complementation group A |

| | | | | |
|---|---|---|---|---|
| FANCD2 | chr3:10068113-10143614 | + | NM_033084 | Fanconi anemia complementation group D2 |
| FBXW7 | chr4:153242410-153456172 | - | NM_033632 | F-box and WD repeat domain containing 7 |
| FGFR1 | chr8:38268656-38326352 | - | NM_015850 | fibroblast growth factor receptor 1 |
| FGFR2 | chr10:123237844-123357972 | - | NM_000141 | fibroblast growth factor receptor 2 |
| FGFR3 | chr4:1795039-1810599 | + | NM_000142 | fibroblast growth factor receptor 3 |
| FGFR4 | chr5:176513921-176525126 | + | NM_002011 | fibroblast growth factor receptor 4 |
| FH | chr1:241660857-241683085 | - | NM_136850 | fumarate hydratase |
| FLT1 | chr13:28874483-29069265 | - | NM_002019 | fms related tyrosine kinase 1, vascular endothelial growth factor receptor 1 |
| FLT3 | chr13:28577411-28674729 | - | NM_004119 | fms related tyrosine kinase 3, CD135 |
| FLT4 | chr5:180028506-180076624 | - | NM_182925 | fms related tyrosine kinase 4, vascular endothelial growth factor receptor 3 |
| FOXA1 | chr14:38059191-38064489 | - | NM_004496 | forkhead box A1 |
| FOXL2 | chr3:138663066-138665982 | - | NM_023067 | forkhead box L2 |
| FTO | chr16:53737875-54148379 | + | NM_001080432 | fat mass and obesity associated |
| GABRA6 | chr5:161112658-161129598 | + | NM_000811 | gamma-aminobutyric acid type A receptor alpha6 subunit |
| GATA1 | chrX:48644982-48652717 | + | NM_002049 | GATA binding protein 1 |
| GATA2 | chr3:128198265-128212030 | - | NM_032638 | GATA binding protein 2 |
| GATA3 | chr10:8096667-8117164 | + | NM_002051 | GATA binding protein 3 |
| GNA11 | chr19:3094408-3121454 | + | NM_002067 | G protein subunit alpha 11 |
| GNAQ | chr9:80335191-80646219 | - | NM_002072 | G protein subunit alpha q |
| GNAS | chr20:57414795-57486250 | + | NM_000516 | GNAS complex locus |
| GSK3B | chr3:119540802-119813264 | - | NM_002093 | glycogen synthase kinase 3 beta |
| H3F3A | chr1:226250408-226259703 | + | NM_002107 | H3 histone, family 3A |
| H3F3B | chr17:73772515-73775860 | - | NM_005324 | H3 histone, family 3B (H3.3B) |
| HIST1H3B | chr6:26031817-26032288 | - | NM_003537 | histone cluster 1, H3b |
| HLA-A | chr6:29910247-29913661 | + | NM_002116 | major histocompatibility complex, class I, A |
| HNF1A | chr12:121416549-121440314 | + | NM_000545 | HNF1 homeobox A |
| HRAS | chr11:532242-535550 | - | NM_005343 | HRas proto-oncogene, GTPase |
| HSP90AB1 | chr6:44214849-44221614 | + | NM_007355 | heat shock protein 90 alpha family class B member 1 |

| | | | | |
|---|---|---|---|---|
| IDH1 | chr2:209100953-209119806 | - | NM_005896 | isocitrate dehydrogenase (NADP(+)) 1, cytosolic |
| IDH2 | chr15:90627212-90645708 | - | NM_002168 | isocitrate dehydrogenase (NADP(+)) 2, mitochondrial |
| IGF1R | chr15:99192761-99507759 | + | NM_000875 | insulin like growth factor 1 receptor |
| IL7R | chr5:35856991-35876923 | + | NM_002185 | interleukin 7 receptor |
| JAK1 | chr1:65298906-65432187 | - | NM_002227 | Janus kinase 1 |
| JAK2 | chr9:4985245-5128183 | + | NM_004972 | Janus kinase 2 |
| JAK3 | chr19:17935593-17958841 | - | NM_000215 | Janus kinase 3 |
| KDM5C | chrX:53220503-53254604 | - | NM_004187 | lysine demethylase 5C |
| KDM6A | chrX:44732423-44971845 | + | NM_001291415 | lysine demethylase 6A |
| KDR | chr4:55944426-55991762 | - | NM_002253 | kinase insert domain receptor |
| KEAP1 | chr19:10596796-10614054 | - | NM_203500 | kelch like ECH associated protein 1 |
| KIT | chr4:55524095-55606881 | + | NM_000222 | KIT proto-oncogene receptor tyrosine kinase |
| KRAS | chr12:25358180-25403854 | - | NM_004985 | KRAS proto-oncogene, GTPase |
| LRP1B | chr2:140988996-142889270 | - | NM_018557 | LDL receptor related protein 1B |
| MAP2K1 | chr15:66679211-66783882 | + | NM_002755 | mitogen-activated protein kinase kinase 1 (MEK1) |
| MAP2K2 | chr19:4090320-4124126 | - | NM_030662 | mitogen-activated protein kinase kinase 2 (MEK2) |
| MAP2K4 | chr17:11924135-12047051 | + | NM_003010 | mitogen-activated protein kinase kinase 4 (MEK4) |
| MAP3K1 | chr5:56110900-56191978 | + | NM_005921 | mitogen-activated protein kinase kinase kinase 1 (MEKK1) |
| MAP3K13 | chr3:185000729-185206882 | + | NM_004721 | mitogen-activated protein kinase kinase kinase 13 (MEKK13) |
| MAP3K4 | chr6:161412822-161538417 | + | NM_005922 | mitogen-activated protein kinase kinase kinase 4 (MEKK4) |
| MAPK1 | chr22:22113947-22221970 | - | NM_002745 | mitogen-activated protein kinase 1 (ERK) |
| MCL1 | chr1:150547027-150552214 | - | NM_021960 | BCL2 family apoptosis regulator |
| MDM2 | chr12:69201971-69239212 | + | NM_002392 | MDM2 proto-oncogene |
| MED12 | chrX:70338406-70362304 | + | NM_005120 | mediator complex subunit 12 |
| MEN1 | chr11:64570986-64578766 | - | NM_000244 | menin 1 |
| MET | chr7:116312459-116438440 | + | NM_001127500 | MET proto-oncogene, receptor tyrosine kinase |
| MITF | chr3:69788586-70017488 | + | NM_198159 | melanogenesis associated transcription factor |
| MLH1 | chr3:37034841-37092337 | + | NM_000249 | mutL homolog 1 |
| MLL | chr11:118307205-118397539 | + | NM_001197104 | KMT2A, histone 3- lysine methyltransferase 2A |

| | | | | |
|---|---|---|---|---|
| MLL2 | chr12:49412758-49449107 | - | NM_003482 | KMT2D, histone 3- lysine methytransferas 2De, mixed lineage leukemia protein-2 |
| MLL3 | chr7:151832010-152133090 | - | NM_170606 | KMT2C, histone 3-lysine methytransferase 2C |
| MPL | chr1:43803475-43820135 | + | NM_005373 | MPL proto-oncogene, thrombopoietin receptor |
| MSH2 | chr2:47630263-47710360 | + | NM_000251 | mutS homolog 2 |
| MSH6 | chr2:48010221-48034092 | + | NM_000179 | mutS homolog 6 |
| MST1 | chr3:49721380-49726196 | - | NM_020998 | macrophage stimulating 1 |
| MST1R | chr3:49924436-49941306 | - | NM_002447 | macrophage stimulating 1 receptor |
| MTOR | chr1:11166588-11322608 | - | NM_004958 | mechanistic target of ra-pamycin |
| MUTYH | chr1:45794914-45806142 | - | NM_012222 | mutY DNA glycosylase |
| MYD88 | chr3:38179969-38184512 | + | NM_001172567 | myeloid differentiation primary response 88 |
| NBN | chr8:90945564-90996899 | - | NM_002485 | nibrin |
| NCOR1 | chr17:15933408-16118874 | - | NM_006311 | nuclear receptor core-pressor 1 |
| NF1 | chr17:29421945-29704695 | + | NM_001042492 | neurofibromin 1 |
| NF2 | chr22:29999545-30094589 | + | NM_000268 | neurofibromin 2 (merlin) |
| NFE2L2 | chr2:178095031-178129859 | - | NM_006164 | nuclear factor, erythroid 2 like 2 |
| NKX2-1 | chr14:36985604-36989430 | - | NM_001079668 | NK2 homeobox 1 |
| NOTCH1 | chr9:139388896-139440238 | - | NM_017617 | notch receptor 1 |
| NOTCH2 | chr1:120454176-120612317 | - | NM_024408 | notch receptor 2 |
| NOTCH3 | chr19:15270444-15311792 | - | NM_000435 | notch receptor 3 |
| NOTCH4 | chr6:32162620-32191844 | - | NM_004557 | notch receptor 4 |
| NPM1 | chr5:170814708-170837888 | + | NM_002520 | nucleophosmin (nucleolar phosphoprotein B23, nu-matrin) |
| NRAS | chr1:115247085-115259515 | - | NM_002524 | neuroblastoma RAS viral oncogene homolog |
| NSD1 | chr5:176560080-176727214 | + | NM_172349 | nuclear receptor binding SET domain protein 1 |
| NTRK1 | chr1:156785542-156851642 | + | NM_002529 | neurotrophic receptor ty-rosine kinase 1 |
| NTRK3 | chr15:88419988-88799962 | - | NM_001012338 | neurotrophic receptor ty-rosine kinase 3 |
| PALB2 | chr16:23614483-23652678 | - | NM_024675 | partner and localizer of BRCA2 |
| PAX5 | chr9:36838531-37034476 | - | NM_016734 | paired box 5 |
| PBRM1 | chr3:52579368-52719866 | - | NM_018313 | polybromo 1 |
| PDCD1 | chr2:242792033-242801058 | - | NM_005018 | programmed cell death 1 |
| PDGFRA | chr4:55095264-55164412 | + | NM_006206 | platelet derived growth factor receptor alpha |
| PDGFRB | chr5:149493402-149535422 | - | NM_002609 | platelet derived growth factor receptor beta |
| PHF6 | chrX:133507342-133562822 | + | NM_032458 | PHD finger protein 6 |

| | | | | |
|---|---|---|---|---|
| PIK3CA | chr3:178866311-178952497 | + | NM_006218 | phosphatidylinositol-4,5-bisphosphate 3-kinase catalytic subunit alpha |
| PIK3CG | chr7:106505924-106547592 | + | NM_002649 | phosphatidylinositol-4,5-bisphosphate 3-kinase catalytic subunit gamma |
| PIK3R1 | chr5:67511584-67597649 | + | NM_181523 | phosphoinositide-3-kinase regulatory subunit 1 |
| PLCG1 | chr20:39766161-39804357 | + | NM_002660 | phospholipase C gamma 1 |
| PMS2 | chr7:6012870-6048737 | - | NM_000535 | PMS1 homolog 2, mismatch repair system component |
| POLE | chr12:133200348-133263945 | - | NM_006231 | polymerase (DNA) epsilon, catalytic subunit |
| PPM1D | chr17:58677544-58743640 | + | NM_003620 | protein phosphatase, Mg2+/Mn2+ dependent 1D |
| PPP1R3A | chr7:113516882-113559082 | - | NM_002711 | protein phosphatase 1 regulatory subunit 3A |
| PPP2R1A | chr19:52693055-52729678 | + | NM_014225 | protein phosphatase 2 scaffold subunit Aalpha |
| PRDM1 | chr6:106534195-106557814 | + | NM_001198 | PR domain 1 |
| PREX2 | chr8:68864603-69143897 | + | NM_024870 | phosphatidylinositol-3,4,5-trisphosphate dependent Rac exchange factor 2 |
| PRG4 | chr1:186265405-186283688 | + | NM_005807 | proteoglycan 4 |
| PTCH1 | chr9:98205264-98279247 | - | NM_001083602 | patched 1 |
| PTEN | chr10:89623195-89728532 | + | NM_000314 | phosphatase and tensin homolog |
| PTK2 | chr8:141668481-142011412 | - | NM_153831 | protein tyrosine kinase 2 |
| PTPN11 | chr12:112856536-112947717 | + | NM_002834 | protein tyrosine phosphatase, non-receptor type 11 |
| PTPRB | chr12:70910632-71031220 | - | NM_001109754 | protein tyrosine phosphatase, receptor type B |
| RAC1 | chr7:6414126-6443598 | + | NM_006908 | ras-related C3 botulinum toxin substrate 1 (rho family, small GTP binding protein Rac1) |
| RAD51 | chr15:40987327-41024356 | + | NM_002875 | RAD51 recombinase |
| RAD51C | chr17:56769963-56811692 | + | NM_058216 | RAD51 paralog C |
| RAF1 | chr3:12625100-12705700 | - | NM_001354689 | Raf-1 proto-oncogene, serine/threonine kinase |
| RARA | chr17:38465423-38513895 | + | NM_000964 | retinoic acid receptor alpha |
| RB1 | chr13:48877883-49056026 | + | NM_000321 | RB transcriptional corepressor 1 |
| RET | chr10:43572517-43625797 | + | NM_020975 | ret proto-oncogene |
| RICTOR | chr5:38938022-39074501 | - | NM_152756 | RPTOR independent companion of MTOR complex 2 |
| RNF43 | chr17:56431038-56494931 | - | NM_017763 | ring finger protein 43 |

| | | | | |
|---|---|---|---|---|
| ROS1 | chr6:117609530-117747018 | - | NM_002944 | ROS proto-oncogene 1, receptor tyrosine kinase |
| RPS6KB1 | chr17:57970443-58027786 | + | NM_003161 | ribosomal protein S6 kinase B1 |
| RPTOR | chr17:78518625-78940173 | + | NM_020761 | regulatory associated protein of MTOR complex 1 |
| RUNX1 | chr21:36160098-36421595 | - | NM_001754 | runt related transcription factor 1 |
| RUNX1T1 | chr8:92967195-93115454 | - | NM_004349 | RUNX1 translocation partner 1 |
| SDHB | chr1:17345225-17380665 | - | NM_003000 | succinate dehydrogenase complex iron sulfur subunit B |
| SDHC | chr1:161284166-161334535 | + | NM_003001 | succinate dehydrogenase complex subunit C |
| SDHD | chr11:111957571-111966518 | + | NM_003002 | succinate dehydrogenase complex subunit D |
| SETBP1 | chr18:42260138-42648475 | + | NM_015559 | SET binding protein 1 |
| SETD2 | chr3:47057898-47205467 | - | NM_014159 | SET domain containing 2 |
| SF3B1 | chr2:198256698-198299771 | - | NM_012433 | splicing factor 3b subunit 1 |
| SMAD2 | chr18:45359466-45457515 | - | NM_005901 | SMAD family member 2 |
| SMAD3 | chr15:67358195-67487533 | + | NM_005902 | SMAD family member 3 |
| SMAD4 | chr18:48556583-48611411 | + | NM_005359 | SMAD family member 4 |
| SMARCA2 | chr9:2015342-2193623 | + | NM_003070 | SWI/SNF related, matrix associated, actin dependent regulator of chromatin, subfamily a, member 2 |
| SMARCA4 | chr19:11071598-11172958 | + | NM_001128849 | SWI/SNF related, matrix associated, actin dependent regulator of chromatin, subfamily a, member 4 |
| SMARCB1 | chr22:24129150-24176705 | + | NM_003073 | SWI/SNF related, matrix associated, actin dependent regulator of chromatin, subfamily b, member 1 |
| SMARCD1 | chr12:50478983-50494494 | + | NM_003076 | SWI/SNF related, matrix associated, actin dependent regulator of chromatin, subfamily d, member 1 |
| SMC1A | chrX:53401070-53449618 | - | NM_006306 | structural maintenance of chromosomes 1A |
| SMC3 | chr10:112327449-112364392 | + | NM_005445 | structural maintenance of chromosomes 3 |
| SMO | chr7:128828713-128853385 | + | NM_005631 | smoothened, frizzled class receptor |
| SOCS1 | chr16:11348274-11350039 | - | NM_003745 | suppressor of cytokine signaling 1 |

| | | | | |
|---|---|---|---|---|
| SOS1 | chr2:39208690-39347604 | - | NM_005633 | SOS Ras/Rac guanine nucleotide exchange factor 1 |
| SOX9 | chr17:70117161-70122560 | + | NM_000346 | SRY-box 9 |
| SPEN | chr1:16174359-16266950 | + | NM_015001 | spen family transcriptional repressor |
| SPOP | chr17:47676246-47755525 | - | NM_001007226 | speckle type BTB/POZ protein |
| SRC | chr20:35973088-36033821 | + | NM_005417 | SRC proto-oncogene, non-receptor tyrosine kinase |
| SRSF2 | chr17:74730197-74733493 | - | NM_003016 | serine and arginine rich splicing factor 2 |
| STAG2 | chrX:123094475-123236505 | + | NM_001042749 | stromal antigen 2 |
| STAT3 | chr17:40465343-40540513 | - | NM_139276 | signal transducer and activator of transcription 3 |
| STK11 | chr19:1205798-1228434 | + | NM_000455 | serine/threonine kinase 11 |
| STK19 | chr6:31938952-31949223 | + | NM_004197 | serine/threonine kinase 19 |
| SUFU | chr10:104263719-104393214 | + | NM_016169 | SUFU negative regulator of hedgehog signaling |
| SYK | chr9:93564012-93660842 | + | NM_003177 | spleen associated tyrosine kinase |
| TBC1D4 | chr13:75858809-76056250 | - | NM_014832 | TBC1 domain family member 4 |
| TBX3 | chr12:115108059-115121969 | - | NM_005996 | T-box 3 |
| TERT | chr5:1253287-1295162 | - | NM_198253 | telomerase reverse transcriptase |
| TET2 | chr4:106067032-106200960 | + | NM_001127208 | tet methylcytosine dioxygenase 2 |
| TGFB1 | chr19:41836812-41859831 | - | NM_000660 | transforming growth factor beta 1 |
| TGFBR1 | chr9:101867412-101916473 | + | NM_004612 | transforming growth factor beta receptor 1 |
| TGFBR2 | chr3:30647994-30735633 | + | NM_001024847 | transforming growth factor beta receptor 2 |
| TNF | chr6:31543350-31546112 | + | NM_000594 | tumor necrosis factor |
| TNFAIP3 | chr6:138188581-138204449 | + | NM_001270508 | TNF alpha induced protein 3 |
| TOP1 | chr20:39657462-39753126 | + | NM_003286 | topoisomerase (DNA) I |
| TOP2A | chr17:38544773-38574202 | - | NM_001067 | topoisomerase (DNA) II alpha |
| TP53 | chr17:7571720-7590868 | - | NM_000546 | tumor protein p53 |
| TSC1 | chr9:135766735-135820020 | - | NM_000368 | tuberous sclerosis 1 |
| TSC2 | chr16:2097990-2138713 | + | NM_000548 | tuberous sclerosis 2 |
| TSHR | chr14:81421869-81612646 | + | NM_000369 | thyroid stimulating hormone receptor |
| U2AF1 | chr21:44513066-44527688 | - | NM_006758 | U2 small nuclear RNA auxiliary factor 1 |
| VEGFA | chr6:43737946-43754223 | + | NM_001025366 | vascular endothelial growth factor A |
| VHL | chr3:10183319-10195354 | + | NM_000551 | von Hippel-Lindau tumor suppressor |

| | | | | |
|---|---|---|---|---|
| WHSC1L1 | chr8:38132561-38239790 | - | NM_023034 | Wolf-Hirschhorn syndrome candidate 1-like 1 |
| WT1 | chr11:32409322-32457081 | - | NM_000378 | Wilms tumor 1 |
| XPO1 | chr2:61705069-61765418 | - | NM_003400 | exportin 1 |
| ZRSR2 | chrX:15808574-15841382 | + | NM_005089 | zinc finger CCCH-type, RNA binding motif and serine/arginine rich 2 |

**Table S3.** Primary and secondary antibodies.

| Primary Antibody | Host/ Isotype | Dilution/ Concentration | Source | Catalog Number |
|---|---|---|---|---|
| anti-RAC1[a] | Rabbit polyclonal IgG | 1:200 | Cell Signaling Technology | 2465 |
| anti-RAC1[b] | Mouse monoclonal IgG | 1:500 | Cell Signaling Technology | 8815 |
| anti-ERK1/2 | Rabbit polyclonal IgG | 1:1000 | Cell Signaling Technology | 4695 |
| anti-pERK1/2 (Thr202/Tyr204) | Rabbit polyclonal IgG | 1:1000 | Cell Signaling Technology | 4370 |
| anti-AKT | Rabbit polyclonal IgG | 1:1000 | Cell Signaling Technology | 4691 |
| anti-pAKT (Thr308) | Rabbit polyclonal IgG | 1:2000 | Cell Signaling Technology | 4056 |
| anti-PAK1 | Rabbit polyclonal IgG | 1:1000 | Cell Signaling Technology | 2602 |
| anti-pPAK1 (Thr423) | Rabbit polyclonal IgG | 1:1000 | Cell Signaling Technology | 2601 |
| anti-TUBA4A | Mouse monoclonal IgG | 1:500 | Sigma-Aldrich | T9026 |
| anti-VCL | Rabbit monoclonal IgG | 1:500 | Cell Signaling Technology | 13901 |
| **Secondary Antibody** | **Host/ Isotype** | **Dilution/ Concentration** | **Source** | **Catalog Number** |
| Mouse IgG | Goat | 1:5000/WB | LI-COR | 926-68070 (680RD) |
| Rabbit IgG | Goat | 1:5000/WB | LI-COR | 926-32211 (800CW) |
| Rat IgG | Goat | 1:5000/WB | LI-COR | 925-68076 (800RD) |
| Mouse IgG | Goat | 1:5000/WB | LI-COR | 925-32210 (800CW) |

[a]Recognizes both RAC1 wild-type and RAC1 (P34R), [b]Recognizes only RAC1 wild-type.

**Table S4.** TaqMan and IDT probes/primers used for RT-qPCR and copy number assays.

**RT-qPCR:**

| Gene | Probe ID |
|---|---|
| *TWIST1* | Hs04989912_s1 |
| *MET* | Hs01565584_m1 |
| *BRAF* | Hs00269944_m1 |
| *EGFR* | Hs01076090_m1 |
| *RAC1* | Hs00251654_m1 |
| *GAPDH* | Hs02758991_g1 |
| *RPLP0* | Hs00420895_gH |

**Copy number assays:**

| Gene | Probe ID |
|---|---|
| | **TaqMan** |
| *RAC1* | Hs04938353_cn |
| *TWIST1* | Hs02813890_cn |
| *EGFR* | Hs07526740_cn |
| *MET* | Hs01432482_cn |
| *BRAF* | Hs04949885_cn |
| *RPPH1* (RNase P, CNV reference assay) | Cat. # 4403326 |

**Table S5.** TaqMan and IDT probes/primers used for RT-qPCR and copy number assays.

**RT-qPCR:**

| Gene | Probe ID |
|---|---|
| *TWIST1* | Hs04989912_s1 |
| *MET* | Hs01565584_m1 |
| *BRAF* | Hs00269944_m1 |
| *EGFR* | Hs01076090_m1 |
| *RAC1* | Hs00251654_m1 |
| *GAPDH* | Hs02758991_g1 |
| *RPLP0* | Hs00420895_gH |

**Copy number assays:**

| Gene | Probe ID |
|---|---|
| | **TaqMan** |
| *RAC1* | Hs04938353_cn |
| *TWIST1* | Hs02813890_cn |
| *EGFR* | Hs07526740_cn |
| *MET* | Hs01432482_cn |
| *BRAF* | Hs04949885_cn |
| *RPPH1* (RNase P, CNV reference assay) | Cat. # 4403326 |
